# Environmental risk assessment related to using resource recovery-based bio-composite materials in the aquatic environment with new laboratory leaching test data

**DOI:** 10.1007/s11356-024-32522-8

**Published:** 2024-02-21

**Authors:** Arianna Nativio, Oriana Jovanovic, Jan Peter van der Hoek, Zoran Kapelan

**Affiliations:** 1 Department of Water Management, Civil Engineering and Geosciences, Delft University of Technology, Stevinweg 1, 2628 CN Delft, The Netherlands; 2 Waternet, Korte Ouderkerkerdijk 7, 1096 AC Amsterdam, The Netherlands

**Keywords:** Bio-composite materials, Canal bank protection environmental risk assessment, Heavy metal contamination, Leaching tests, Sensitivity analysis, Surface water

## Abstract

The concept of circular economy, aiming at increasing the sustainability of products and services in the water and other sectors, is gaining momentum worldwide. Driven by this concept, novel bio-composite materials produced by recovering resources from different parts of the water cycle are now manufactured in The Netherlands. The new materials are used for different products such as canal bank protection elements, as an alternative to similar elements made of hardwood. As much as these new materials are appealing from the sustainability point of view, they may leach toxic substances into the aquatic environment given some of their ingredients, e.g., cellulose recovered from wastewater treatment. Therefore, a methodology for the assessment of related environmental risks is needed and it does not exist currently. This paper addresses this knowledge gap by presenting a framework for this. The framework is based on European environmental risk assessment guidelines, and it includes four key steps: (i) hazard identification, (ii) dose–response modelling, (iii) exposure assessment and (iv) risk characterisation (i.e. assessment). As part of the first step, laboratory leaching tests were carried out to evaluate the potential release of specific chemical substances such as heavy metals and resin compounds into the aquatic environment. Laboratory test results were then used as input data to evaluate the risk of potential leaching from canal bank protection elements into surface water. A deterministic model was used first to identify the chemicals exceeding the guideline threshold. Subsequently, a stochastic model was applied to evaluate the environmental risks across a range of leachate concentrations and water velocities in the canal, thereby simulating a broader spectrum of possible situations. The risk analyses were conducted for four alternative bio-composite materials made of different ingredients, two different flow conditions (stagnant water and advective flow) in two types of canals (wide ditch and primary watercourse) and for two different water levels based on season conditions (summer and winter conditions). The results obtained from leaching tests identified Cu, Mn, Zn, styrene and furfuryl alcohol as potentially troublesome chemicals. In the case of stagnant water, the absence of a flow rate increases the residence time of the chemicals in the surface water, resulting in a higher PEC/PNEC (i.e. risk) value. However, under stagnant case conditions, environmental risks for all chemicals considered turned out to be below the safety threshold. In the advective case, the existence of a flow rate, even at low velocities simulating the conditions of ‘almost no flow,’ contributes to increased dilution, resulting in lower PEC/PNEC ratio values. The results presented here, even though representing real-case scenarios, are only indicative as these are based on laboratory leaching tests and a number of assumptions made. Additional field tests involving collecting and analysing water and sediment samples from the canal where the canal bank protection elements are located, over a prolonged period, are required to come up with more conclusive findings.

**Supplementary Information:**

The online version contains supplementary material available at 10.1007/s11356-024-32522-8.

## Introduction

The move towards circular economy solutions is desired and encouraged for several reasons, including the availability and low cost of upcycled products and economic well-being. The world’s population is growing and with it the demand for raw materials, while the availability of raw materials is limited. The use of upcycled materials, according to European reports (E.U.C, 2023), may reduce the use of natural resources, reduce landscape and habitat disruption and help limit biodiversity loss. Another reason for moving to a circular economy is the potential to reduce greenhouse gas emissions (E.U.C, 2023). Creating sustainable products based on recovered and recycled materials helps to reduce energy and resource consumption.

In The Netherlands, novel bio-composite materials have been successfully developed by using various resources recovered from different parts of the water management cycle (Nativio et al. 2022). These bio-composite materials are used to replace traditional materials such as hardwood in the manufacture of a range of products, including canal bank protection elements. As these materials and related products are produced by recovering resources from wastewater and other sources and using resins, there is a potential for toxic substances such as heavy metals (e.g. Co, Cu, Cr, Pb, V, Zn and As) and resin compounds to leach into the environment. Contamination with heavy metals is a pervasive phenomenon in nature, demonstrable by the prominent accumulation of aluminium in indoor environments as reported by Cetin and Abo Aisha (2023). Furthermore, a comprehensive investigation conducted by Cetin et al. (2020) evaluated heavy metal pollution specifically focusing on the assessment of calcium, copper and lithium contamination in blue spruce trees. Complementing this research, Cesur et al. (2021) investigated the accumulation of heavy metals in plant organs. The trend of accumulation of heavy metals poses concern in terms of air and soil pollution. In line with the latter, bio-monitors, such as trees, soil and plants, commonly serve as effective tools for monitoring atmospheric heavy metal concentrations. Cetin et al. (2022) conducted also an insightful study mapping the accumulation patterns of specific heavy metals, including nickel and cobalt, through topsoil sampling.

In the context of the current study, it is crucial to evaluate the applicability of novel materials as canal bank protection elements and the potential hazards associated with the water body contamination. The presence of heavy metals poses substantial concerns for both surface and groundwater quality, given their documented adverse effects on aquatic ecosystems, agriculture and human health, as demonstrated in previous studies (Naito et al. 2010; Srinivasa Gowd & Govil 2007). Consequently, it is essential to evaluate the environmental risks related to the use of these new materials in aquatic environments.

Currently, there are no established industry standards for the environmental risk assessment of novel bio-composite materials, regardless of their application. We propose a methodology based on European environmental risk assessment guidelines (E.C.H.A., 2023; Manuilova 2003; van Vlaardingen et al. 2005) that uses an experimental approach applied under relevant conditions, taking into account the allowable environmental limits. For this purpose, the viability of laboratory leaching tests is evaluated based on published literature. The value of conducting leaching tests for environmental risk assessment has been demonstrated previously by van der Sloot et al. (2017). They described how leaching tests can be used to conduct an environmental risk assessment for construction materials. Leaching tests have been instrumental in characterising soil contamination with potential implications for underground water quality, as highlighted by Amlal et al. (2020). These tests offer valuable insights into leaching rates, chemical accumulation and associated risks. Kabiri et al. (2022) conducted diverse leaching test methods to explore PFASs leaching behaviour from soil. Additionally, leaching tests were employed by Guleria and Chakma (2019) for human health risk assessment, specifically evaluating heavy metals leaching to assess potential risks via dermal contact. Notably, all the mentioned studies in the literature employed leaching tests based on a simulation approach. The latter aims to replicate specific field scenarios in laboratory settings, ensuring the results are interchangeable and comparable.

The goal of carrying out the environmental leaching tests is to provide an estimation of the leaching potential of constituents from various materials across a range of potential scenarios. Therefore, selection of the appropriate leaching test and testing protocol is crucial for the reproducibility and downstream usability of the results. Four internationally recognised types of leaching tests are the pH-dependence test, the percolation test, the monolith test and the compacted granular test (van der Sloot et al. 2017). In the pH-dependence test, solid materials are submerged into eluents of different pH for fixed time intervals. Variation of a single parameter (pH) captures the response of the material to different environmental conditions (acidic/basic). The percolation test, or as often referred to in the literature as dynamic column leaching test, evaluates the leaching of the material as a function of eluent volume and dry weight of the material. The monolith test is used for the analysis of single solid pieces of material. From this method, information about the predominant release mechanism can be obtained. Leaching of the finely grounded clays is analysed with a compacted granular test. This test is performed in the same manner of as the monolith leaching test, but applicable only for fine-grained materials. van der Sloot and Kosson (2012) performed a comparative analysis between the pH-dependence leaching test and percolation (column leaching test) test, for the leaching from hazardous waste and industrial sludge. At the same pH and L/S (liquid/solid) ratio, the results from both tests are comparable. However, if the material is expected to be exposed to the eluents with the range of pH values, then the pH-dependence tests would provide a more comprehensive picture.

Abovementioned leaching tests and other tests documented in the literature (van der Sloot & Kosson 2012; van der Sloot et al. 2017) show that tests can be conducted across a spectrum of scenarios involving different materials (waste, ash, construction materials, soil, sediments, etc.) and water samples (Bridson et al. 2021). The choice of the specific type of leaching test to be undertaken should be guided by the objectives of the study and the chemicals to be analysed (Bridson et al. 2021). In this study, the percolation dynamic column leaching test was utilised. This test was selected according to the scenario to be simulated. The aim of this study is to analyse the leaching of heavy metals and resin compounds such as styrene and furfuryl alcohol, from the novel bio-composite materials used as canal bank protection and their potential impact on the aquatic environment. The environmental scenario is therefore characterised by surface water with an almost constant pH and continuous flow rate. This excludes the use of pH-dependence tests. The monolithic test is used to evaluate the mass transfer of monolithic construction materials such as concrete.

In this study, a new type of bio-composite material made from resources recovered from the water sector was analysed. Thus, the percolation column leaching test (column leaching test) was selected as more appropriate for the purpose of this study and for the environmental conditions to be simulated.

The leaching of the chemicals depends on several factors. As demonstrated by van der Sloot and Kosson (2012), chemical parameters, such as pH of the eluent, redox potential, kinetics, adsorption, ion exchange and electrostatic attraction, affect the leaching behaviour. Cappuyns and Swennen (2008) assessed the mobility of heavy metals within river soil sediments, depending on the eluent pH. The mobility of elements such as Zn, Cd, Ni and Mn increased at decreasing pH, for others such as Cu and Fe, a higher leachability was observed at greater pH values. From these studies, it can be concluded that variations in the pH can greatly affect the results. As mentioned above, the environmental scenario consists of the application of these novel materials as canal bank protection in surface water canals. Therefore, no significant pH variations are expected. In order to remove the effects of pH changes on the results, in this study, it was decided to use buffered ultrapure water having a constant pH of 7.00 ± 0.2 as influent.

Oppel et al. (2004) investigated the environmental impact of pharmaceutical leaching in soil and groundwater and the consequent toxicity to human health due to groundwater contamination. Their study highlights that examining the environmental impacts of a product in the aquatic environment may have implications for human health and safety, resulting in impacts on the entire ecosystem. In this study, only the environmental risk associated with the use of these materials in an aquatic environment was assessed. This was done because of the novelty of these new bio-composite materials. These materials have never been tested for environmental risk assessment and have never been used for canal bank protection or other applications yet.

The majority of the research reported in the above literature utilised soil and/or sediment samples for their studies. On-site samples were collected to assess potential leaching, after which laboratory leaching tests were employed to replicate leaching under controlled conditions. Subsequently, the obtained results were compared with chemical analyses of on-site leaching samples, serving as a baseline for environmental risk assessment. However, in the current study, collecting on-site samples as a baseline for laboratory leaching tests was not feasible; thus, only laboratory leaching tests were performed to assess the environmental risks. On the basis of the above, a knowledge gap was identified: no environmental risk assessment of the leaching of toxic substances into the aquatic environment from these new bio-composite materials, particularly those used as canal bank protection elements and similar products, has been carried out. By conducting leaching tests under controlled laboratory conditions, it may be possible to obtain a more complete general picture of constituents leaching from different bio-composite materials. In the meantime, these data provide significant information that could be used for environmental risk assessment. In the absence of a baseline, usually represented by on-site water and sediment samples, a number of assumptions had to be made, as described in ‘‘Materials and methods’’ of this paper. This, in turn, has led to the selection of the existing environmental risk assessment (ERA) framework (Manuilova 2003) for environmental risk analysis.

In this paper, the fate of chemicals once leached from the canal bank protection element, made of bio-composite material, into the surface water was not part of the scope of this research. However, it would be interesting to extend the framework with artificial neural network modelling to predict contaminant behaviour, and subsequent concentrations, in surface water. Moreover, artificial neural network modelling can be utilised to model the adsorption of heavy metals into the soil and monitor their fate within the groundwater cycle (Ucun Ozel et al. 2020).

The paper is structured as follows. The experimental setup and the methodology are described in ‘‘Materials and methods’’. The results for both a deterministic and stochastic risk assessment approach are presented in ‘‘Results and discussion’’. Conclusions are then presented in ‘‘Conclusions’’.

## Materials and methods

The identified knowledge gap concerning the leaching test and the environmental impact assessment of these emerging bio-composite materials, particularly in water environments, have been addressed by proposing a framework. This framework is based on three building blocks: (i) the existing environmental risk assessment (ERA) (Manuilova 2003) and the corresponding Dutch guidelines published by the Dutch National Institute for Public Health and the Environment (RIVM) (van Vlaardingen et al. 2005); (ii) the database of European Chemicals Agency (E.C.H.A., 2023); (iii) percolation column leaching tests (U.S.E.P.A. 2017) providing input data for the environmental risk assessment, and simulating the real case scenario in absence of detailed on-site data due to the novelty of these materials. Leaching test results were used and modelled to simulate the potential leaching in the real canal under various environmental conditions such as a stagnant case (absence of flow rate) and the presence of flow rate. Two types of canals were analysed. No other leaching tests were performed on this new type of bio-composite material up till now, while the corresponding environmental risk analysis was not conducted so far.

### Bio-composite materials and their use for canal bank protection elements

This work assesses the environmental risks associated with potential leaching of heavy metals and resin compounds from canal bank protection elements made from new bio-composite materials. The purpose of canal bank protection elements is to protect the canal bank from soil collapse into the water. The canal bank elements are placed on both sides of the canal. Four different alternatives of new bio-composite materials (M1, M2, M3 and M4) are considered to produce these elements. Table 1 presents the raw components used in the four alternatives bio-composite materials. The four materials are all made of natural fibres and fillers (e.g. water reeds, wastewater cellulose and bio-filler from agricultural waste) with additives added for different purposes, and all bonded together using a resin (e.g. polyester resin or furan resin).

**Table 1:** Bio-composite materials used for canal bank protection elements

M1	M2	M3	M4
Water reeds	Water reeds	Wastewater cellulose	Grass
Mined calcite	Calcite mix^1^	Calcite mix	Bio-filler from agricultural waste
Polyester resin^2^	Unsaturated polyester resin with 50% bio-content	Unsaturated polyester resin with 50% bio-content	Furan resin^3^
Additives^4^	Additives	Additives	Additives

^1^Calcite mix: mix of 50% mined calcite and 50% recycled calcite from drinking water softening process. ^2^Polyester resin, as well as unsaturated polyester resin with 50% bio-content contain styrene. ^3^Furan resin containing furfuryl alcohol. ^4^Additives: used as catalyst to stimulate the chemical reaction (polymerisation) of the resin, improve impact resistance (impact agents), release agents to prevent sticking to the mould and allow releasing the part (release agents)

**Fig. 1:**
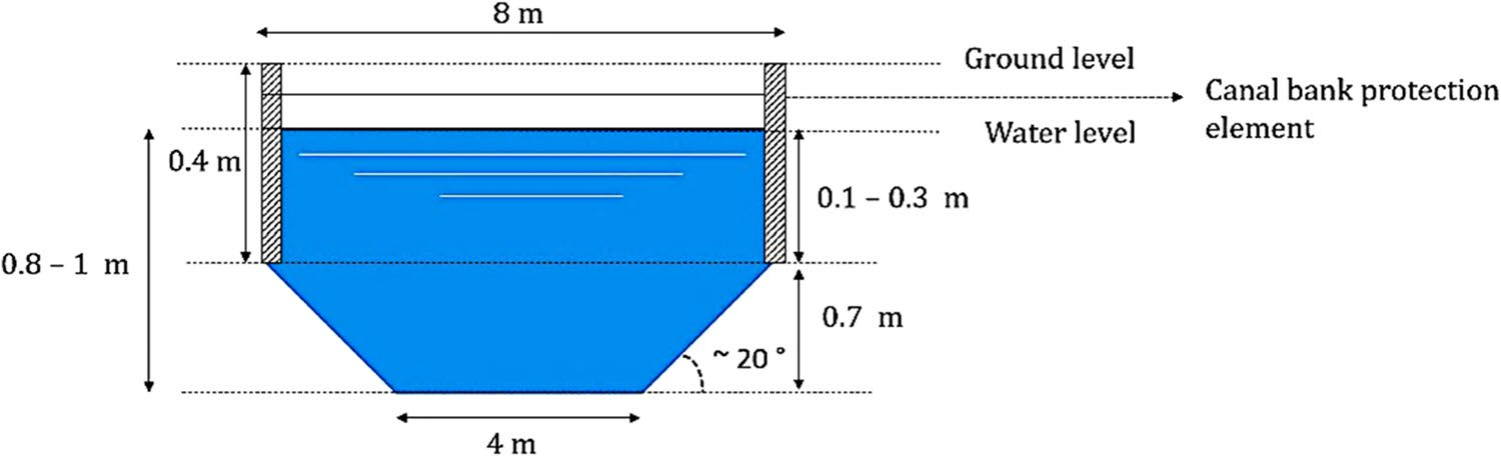
Wide ditch profile

The analysed water canal system consists of (i) wide ditch and (ii) primary watercourse. To estimate the flow rate, the full operation of the nearby pumping station, located along the primary watercourse, was taken into account. Furthermore, the flow rate varies based on the water level, which varies according to the seasonal conditions (summer and winter conditions). The characteristics of the wide ditch and primary watercourse are as follows (see Fig. 1 for the wide ditch and Fig. 1 in the Supplementary Material for the primary watercourse).

1. Wide ditch: trapezoidal profile, bottom width of 4 m, top width of 8 m and effective depth of 1 m in the middle. The water cross-section velocity was measured and found to have an average of 2.4 m/h. The flow rate based on water cross-section area was calculated to have an average value of 11.52 m^3^/h under summer conditions (low water level due to the dry season). In contrast to an average value of 14.4 m.^3^/h, under the winter conditions (high water level due to the rainy season)
2. Primary watercourse: similar profile as the wide ditch (trapezoidal profile) with a bottom width of 5 m and a top width of 10 m, and a depth of 1 m in the middle. The water cross-section velocity was measured to be 270 m/h on average. The flow rate, based on the water cross-sectional area, was estimated to be 1620 m^3^/h under summer conditions (low water level due to the dry season) and 2025 m.^3^/h under the winter conditions (high water level due to the wet season)

The pumping station works approximately 800 h per year.

The canal bank protection elements, placed on both sides of the canal, are of conventional pile-bulkhead construction, using a combination of softwood where possible (always below the waterline), and bio-composites where needed to extend the lifespan of the system (below and above the waterline, depending on the season). The water level changes with the seasons, as explained above. Based on expert knowledge, it was assumed that the water level varies by 0.2 m between the summer and winter seasons. The 0.4 m high bio-composite elements are then assumed to be submerged by a minimum of 0.1 m (low water level representing summer season conditions) to a maximum of 0.3 m (high water level representing winter season conditions). Only the submerged part of the bio-composite bank protection elements is considered in this paper.

Table 2 lists the mass of the submerged bio-composite material over 1 m of length, during the summer and winter season conditions. The dimensions of the canal bank protection elements (e.g. width, length and thickness) are the same for all four bio-composite alternatives. Each bio-composite alternative has a specific density, which defines the actual submerged mass. Density data are not shown here for confidentiality reasons.

**Table 2:** Mass of submerged bio-composite material during the summer and winter season conditions per 1 m of length of canal bank protection

Material	Flow regime	Mass of submerged material per m of canal length (kg/m)
M1	Summer conditions	1.032
	Winter conditions	3.096
M2	Summer conditions	1.032
	Winter conditions	3.096
M3	Summer conditions	1.068
	Winter conditions	3.204
M4	Summer conditions	0.828
	Winter conditions	2.484

### Column leaching test

**Fig. 2:**
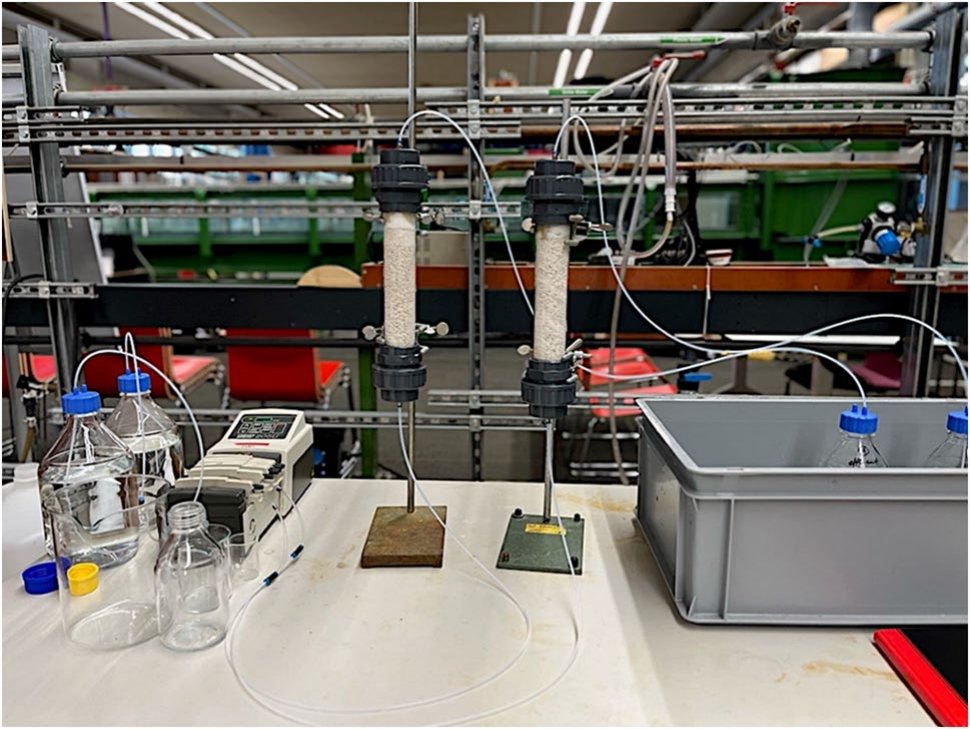
Experimental setup for leaching tests

To analyse the potential leaching of heavy metals and resin compounds from bio-composite materials in an aquatic environment, percolation column leaching tests were carried out, following Dutch standard Guidelines (N.E.N.-7373 2004) and U.S.E.P.A. Method 1314 (U.S.E.P.A. 2017). Percolation test, called column leaching test in this paper, is a dynamic leaching test method that requires the continuous renewal of the influent. Column leaching test method was selected based on the purpose of the study: evaluate the potential leaching from new bio-composite canal bank protection in surface water (canals at almost constant pH and various flow rates). As mentioned in ‘‘Introduction’’, Bridson et al. (2021) suggested to select carefully the leaching test method based on the environmental conditions. In the present study, canal bank protection elements, submerged in surface water canals, were analysed in terms of their potential leaching. Thus, a test that took into account a constant renewal of the influent, by using a pump providing fresh influent continuously to the columns, was preferable. Figure 2 shows the experimental setup.

The bio-composite materials were grinded to a particle size up to 4 mm and packed in sealed glass columns (0.3 kg of material in each column) to a height of 20 cm. Gauzes and quartz sand (20–30 mesh) were used as filters at the bottom and the top of the columns. A buffered ultrapure water at pH 7.00 ± 0.2 was used as influent for the experiments. The columns were operated at a specific liquid–solid ratio (L/S). The liquid-to-solid ratio is defined as the fraction of the total liquid volume (including the moisture contained in the ‘as-used’ solid sample) to the dry mass equivalent of the solid material. L/S is typically expressed in volume units of liquid per dry mass of solid material (ml/g-dry) (U.S.E.P.A. 2017). A flow rate of 9 ml/h was chosen to maintain the liquid–solid ratio between 0.5 and 1.0 l/kg per day, specifically about 0.72 l/kg in this case, to facilitate the higher probability of achieving local equilibrium between the solid and liquid phase (U.S.E.P.A. 2017). The effluent samples to be analysed were selected on the basis of the liquid–solid ratio as specified in the leaching tests standard and guidelines (N.E.N.-7373 2004; U.S.E.P.A. 2017), as shown in Table 3. Four 24 h composite samples (shown in bold in Table 3) were collected on days 1, 2, 6 and 13 of the 2-week periods for which the leaching tests lasted. The effluent samples were collected and stored in the fridge at 4 °C, until further analysis. Analyses for resin compounds (styrene and furfuryl alcohol) were performed within 1 week of sampling as both styrene and furfuryl alcohol are VOCs (volatile organic compounds). All leaching tests were carried out in duplicates. ICP-MS was used to measure the concentrations of heavy metals in water samples. The samples were homogenised and acidified (HNO_3_), after which the analysis was performed from the liquid phase. GC–MS was used to measure styrene in water samples. HPLC–UV was used for measuring furfuryl alcohol in the samples.

**Table 3:** Effluent fractions sampling based on NEN7373, NEN7343 and USEPA method 1314 guidelines

Fraction	Volume (l)	S (kg)	L/S in l/kg
1	0.22	0.3	0.72
2	0.43	0.3	1.44
3	0.65	0.3	2.16
4	0.86	0.3	2.88
5	1.08	0.3	3.60
6	1.30	0.3	4.32
7	1.51	0.3	5.04
8	1.73	0.3	5.76
9	1.94	0.3	6.48
10	2.16	0.3	7.20
11	2.38	0.3	7.92
12	2.59	0.3	8.64
13	2.81	0.3	9.36

### Environmental risk assessment

**Fig. 3:**
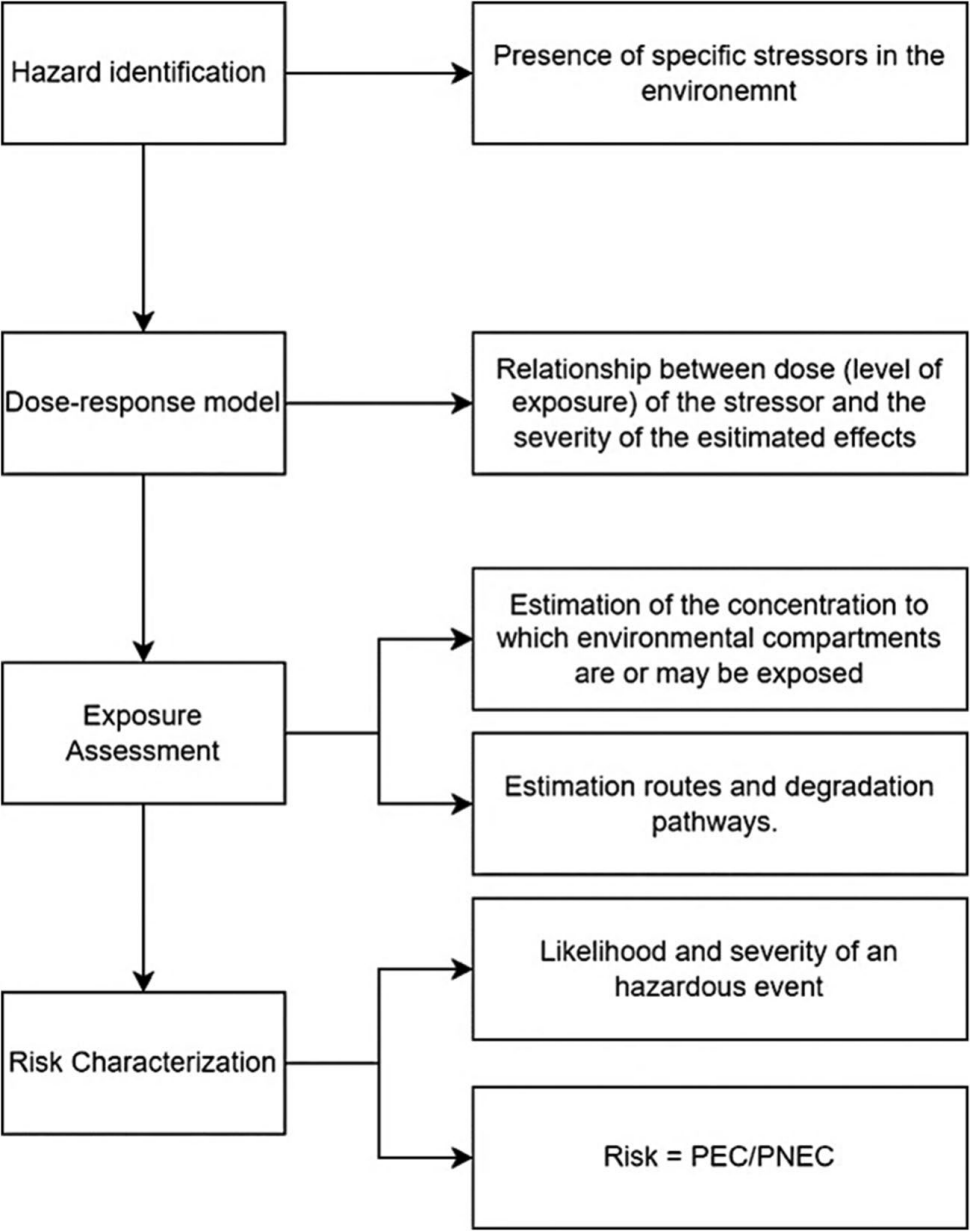
Scheme of the environmental risk assessment based on the European and RIVM Dutch Guidelines

The environmental risk assessment framework used in this study (Manuilova 2003) is based on European guidelines and the RIVM Dutch guidelines (van Vlaardingen et al. 2005) and shown in Fig. 3.

Environmental risk assessment (ERA) is a method used to evaluate the environmental safety of manufactured products, focusing on the potential impacts of pollutants on ecosystems, including various species and the long-term consequences of contaminant releases. The ERA framework, based on European guidelines, categorises environmental compartments such as seawater, freshwater and soil and identifies affected species such as fish, algae and microorganisms.

The ERA framework involves the following four main steps:

1. Hazard identification: Presence of heavy metals and resin compounds in the leaching effluents
2. Dose–response model: Freshwater (surface water in which the canal bank protection elements are applied) is selected as the receiving ecosystem. The predicted no effect concentrations (PNEC) for freshwater were collected from European Chemical Agency (ECHA) database (E.C.H.A., 2023). The PNEC represents the concentration of a substance below which adverse effects are unlikely to occur in both long-term and short-term exposure scenarios. Table 4 shows the PNEC values in [μg/l]
3. Exposure assessment: The predicted effects concentration (PEC) is the predicted environmental concentration of the contaminant. The next step is to assess whether the pollutant can pose a threat at this concentration. The PECs have been estimated for various environmental conditions, which are explained in ‘‘Leaching scenarios’’. The input data for this exposure assessment were derived from the laboratory leaching tests. These data were used to calculate the potential concentrations in the real case scenarios, taking into account key factors such as the dimensions of the bank protection and the canal, as well as the flow rates (‘‘Leaching scenarios’’)
4. Risk characterisation: the environmental risk was calculated as follows:
  1 $${\text{Risk}}=\frac{{\text{PEC}}}{{\text{PNEC}}}$$

**Table 4:** List of PNEC values from the ECHA database

Chemical	PNEC [μg/l]
Mercury (Hg)	0.057
Barium (Ba)	114.7
Boron (B)	2900
Cobalt (Co)	1.06
Copper (Cu)	6.3
Lithium (Li)	1650
Manganese (Mn)	34
Molybdenum (Mo)	11,900
Tin (Sn)	37
Vanadium (V)	4.1
Zinc (Zn)	14.4
Cadmium (Cd)	0.19
Chromium (Cr)	6.5
Nickel (Ni)	20
Lead (Pb)	2.4
Arsenic (As)	5.6
Styrene (C_8_H_8_)	28
Furfuryl alcohol (C_5_H_6_O_2_)	170

When the PEC/PNEC ratio is below the threshold of 1.00 set by the European and Dutch guidelines, the risk level is considered acceptable (Manuilova., 2003; van Vlaardingen et al. 2005). Exceedance of the threshold of 1.00 means that the PEC is greater than the PNEC and is not acceptable.

The above ERA methodology was conducted using the following assumptions:

1. No further degradation or transformation of the leached chemicals occurs in the canal
2. Leached chemicals are instantaneously mixed with canal water and no Brownian motion occurs

### Leaching scenarios

Two case studies, stagnant water and advective flow, were analysed to simulate different real-world flow conditions. These two cases were analysed each for two different types of canals (wide ditch and primary watercourse, described in ‘‘Bio-composite materials and their use for canal bank protection elements’’), and for winter and summer conditions resulting in a total of eight cases. The description of the case studies (stagnant water and advective flow) is given below.

1. Stagnant water case: when the pumping station is off, the scenario assumes instantaneous mixing and no Brownian motion. Both summer and winter conditions were evaluated for the wide ditch and the primary watercourse. Contaminant concentrations in the water, based on leaching from the canal bank protection elements, were calculated for all four bio-composite materials
2. Advective flow case: the pumping station operates 800 h per year. Both the wide ditch and the primary watercourse were evaluated under summer and winter conditions. The contaminant concentrations in the water, based on leaching from the canal bank protection elements, were calculated for all four bio-composite materials

The environmental risk was assessed using both a deterministic approach and a stochastic approach (using the Monte Carlo method) in all 8 cases mentioned above. The deterministic approach was used to assess whether one or more chemicals exceeded the safety threshold. The stochastic approach was used to perform a sensitivity analysis. This was done by using the Monte Carlo method to evaluate how the environmental risk varies with different input data such as leachate concentration and water velocity. Two sensitivity cases were analysed.

1. Sensitivity s1 case: simulation of leachate concentrations using a uniform distribution based on leaching test results. Two batches of data were collected from the duplicate tests: one from column 1 (effluent 1) and one from column 2 (effluent 2) for day 1. The objective of this sensitivity analysis was to assess how the environmental risk level varies with different leachate concentrations
2. Sensitivity s2 case: various water velocities and consequently various flow rates were considered, ranging from a minimum value of 0.2 m/h (representing the ‘almost no flow conditions’) to a maximum value of 270 m/h corresponding to the pumping station in full operation on the primary watercourse. The water velocity was simulated using a normal distribution, with the mean and standard deviation calculated geometrically based on the generated vector. The objective of this scenario was to evaluate how the environmental level varies with flow rates and flow velocities

To simulate the real-world conditions, leaching test data were used as input to predict the potential release of chemicals in the real case for both the wide ditch and primary watercourse and for both the stagnant and advective scenario, taking into account the seasonal variation in flow rate. First, the mass of contaminants released per day under laboratory leaching test conditions was calculated. The equivalent mass released from the submerged bio-composite canal bank protection elements was then calculated. These calculations considered both winter and summer conditions as shown in the following equations:

2 $${M1}_{s}={C}_{L}*{{\text{Vol}}}_{L}*\frac{{M}_{{\text{sub}},s}}{{M}_{L}}$$

3 $${M1}_{w}={C}_{L}*{{\text{Vol}}}_{L}*\frac{{M}_{{\text{sub}},w}}{{M}_{L}}$$

where:

- M1_*s*_ and M1_*w*_ are the masses released over 1 m of canal bank from both sides in one day during the summer conditions (${M1}_{s}$) and winter conditions (${M1}_{w}$), respectively [mg]
- *C*_*L*_ is the concentration from the laboratory leaching test at day 1 [mg/l]
- Vol_*L*_ is the effluent volume of the leaching tests in 24 h [l]
- *M*_sub,*s*_ and *M*_sub,*w*_ are the mass of the bio-composite submerged in the water [kg], over 1 m of canal length, at both sides, during the summer conditions (${M}_{{\text{sub}},s}$) and winter conditions (${M}_{{\text{sub}},w}$), respectively, listed in Table 2
- *M*_*L*_ is the mass of the material used in the leaching test (0.3 kg for each analysed bio-composite alternative)

${M1}_{s}$ and ${M1}_{w}$ were then used as input to calculate the actual leachate concentrations firstly for the stagnant water scenario and then for the advective flow scenario. The concentrations for both summer and winter conditions for both stagnant and advective cases are given by Eqs. 4, 5, 6 and 7, respectively.

4 $${C1}_{stagn,s}=\frac{{M1}_{stagn,s}}{{{\text{Vol}}}_{w,s}}$$

5 $${C1}_{stagn,w}=\frac{{M1}_{stagn,w}}{{{\text{Vol}}}_{w,w}}$$

6 $${C1}_{Q,s}=\frac{{M1}_{stagn,s}}{{{\text{Vol}}}_{w,s}*{F}_{s}}$$

7 $${C1}_{Q,w}=\frac{{M1}_{stagn,w}}{{{\text{Vol}}}_{w,w}*{F}_{w}}$$

where:

- C1_*stagn,s*_ and C1_*stagn,w*_ are the concentrations that will be present in the canal compartment of 1 m length due to the leaching under stagnant case, for summer conditions (${C1}_{stagn,s}$) and winter conditions (${C1}_{stagn,w}$), respectively [mg/m^3^]. These concentrations are then expressed in µg/l for comparison with the PNEC values collected for the freshwater compartmentwhere $Q$ is the flow rate in the canal in [m^3^/day] and *V* is the volume of water [m^3^] for 1 m length of canal.
- M1_*stagn,s*_ and M1_*stagn,w*_ are the released concentrations from the canal banks in one day calculated using Eqs. 2 and 3 [mg] under stagnant case for summer (${M1}_{stagn,s}$) and winter (${M1}_{stagn,w}$), respectivelywhere $Q$ is the flow rate in the canal in [m^3^/day] and *V* is the volume of water [m^3^] for 1 m length of canal.
- Vol_*w,s*_ and Vol_*w,w*_ are the volumes of the water per 1 m of canal length, dependent on the water level in summer conditions (${{\text{Vol}}}_{w,s}$) and winter conditions (${{\text{Vol}}}_{w,w}$), respectively [m.^3^]where $Q$ is the flow rate in the canal in [m^3^/day] and *V* is the volume of water [m^3^] for 1 m length of canal.
- *C*1_*Q,s*_ and *C*1_*Q,w*_ are the concentrations that will be present in the canal compartment of 1 m length due to leaching under advective flow conditions for both summer (${C1}_{Q,s}$) and winter (${C1}_{Q,w}$) conditions, respectively [mg/m^3^]. These concentrations are then expressed in µg/l for comparison with the PNEC values collected for the freshwater compartmentwhere $Q$ is the flow rate in the canal in [m^3^/day] and *V* is the volume of water [m^3^] for 1 m length of canal.
- *F*_*s*_ and *F*_*s*_ are factors that represent the number of times the volume of water is renewed under summer (${F}_{s}$) and winter (${F}_{w}$) conditions in 1 m compartment per day. The factor *F* is calculated as follows:where $Q$ is the flow rate in the canal in [m^3^/day] and *V* is the volume of water [m^3^] for 1 m length of canal.
  8 $$F=\frac{Q}{V}$$

## Results and discussion

### Leaching tests

**Fig. 4:**
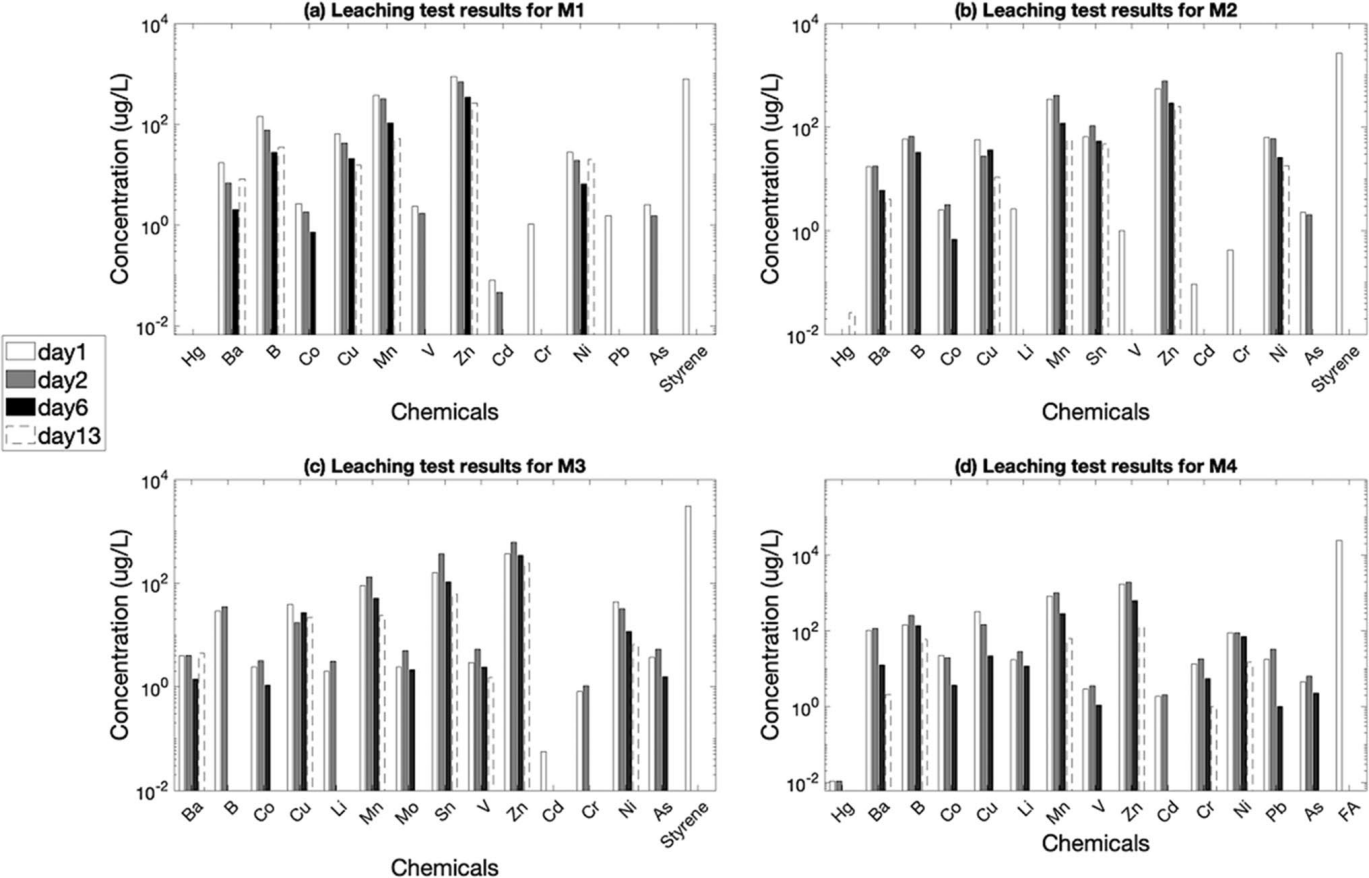
Leaching test results for all four bio-composite materials

The leaching test results for four different bio-composite materials (M1–M4) are shown in Fig. 4.

As can be seen from Fig. 4, some heavy metals such as Co, Cu, Mn, Sn, Zn, Cd, Cr, Ni and Pb leached from all four materials at significantly high concentrations ranging from 100 to 10,000 µg/l. In addition, styrene (in M1, M2 and M3) and furfuryl alcohol (in M4) were identified as resin compounds leaching from the bio-composite materials with the highest leachate concentrations up to 10,000 µg/l. In the column tests, the highest releases were observed at L/S ratio of 0.2 L/kg on day 1 and 0.5 L/kg on day 2, at a pH level 7.00 ± 0.2. These results are aligned with the findings reported by van der Sloot and Kosson (2012).

As shown in Fig. 4, the concentration of various heavy metals such as Ba, Cu, Mn, Zn, Cr, Ni, Pb and As in the leachate was higher on the second day than on the first day, for all four bio-composite materials. After 24 h, heavy metals could potentially be released in higher amounts compared to the leachate concentrations observed on day 1. Several factors influence the leaching behaviour, such as variability of pH of the effluent or the intensity of the flow rate. Cappuyns and Swennen (2008) conducted research on the mobility of heavy metals due to the leaching, as described in ‘‘Introduction’’ of this paper. However, in the leaching tests performed here on new bio-composite materials, the pH remained constant due to the consistent influent characteristics (buffered ultrapure water with a pH of 7.00 ± 0.2). No acidification of the samples was introduced to replicate various environmental conditions such as rainfall events. The collection of effluent samples, pH measurements were consistently monitored. The monitoring results obtained showed a near constant pH value between 6.8 and 7.2. Therefore, the observed leaching behaviour cannot be related to pH variation but is likely to be related to other factors such as slow flow rate, amount of contaminant in the bio-composite materials analysed and freshness of the materials. Details of the observed leaching behaviour are discussed below.

As it can be seen from Fig. 4, the observed gradual dissolution of metal compounds is subject to a time delay. The delay may be attributed to the low infiltration rate resulting from a slow flow rate (set at 9 ml/h). The reduced infiltration rate causes the water to move slowly through the solid material, resulting in a gradual release of the metals over time rather than an immediate response. The delay was observed only at day 2; then, the leachate concentrations showed a gradual decrease until day 13, as expected.

Another factor that can influence leaching was observed to be the freshness of the samples tested. The bio-composite materials M1, M2 and M3, although containing a similar amount of polyester resin, showed a significant difference in styrene leaching, as shown in Fig. 4a–c representing the leaching of M1, M2 and M3, respectively. This discrepancy in styrene leachate concentration could possibly be attributed to the freshness of the materials, knowing that styrene is a volatile organic compound. These observed results are in line with the findings of Dandautiya et al. (2018), that in their study analysed a fresh and a weathered sample 30 days after its disposal of fly ash. The aged material was not representative of the long-term effects (weathered of 30 days) who observed a lower leaching in the weathered samples compared to the fresh samples even if the authors analysed a short-time age difference such as 30 days. In the present study, materials M2 and M3 were analysed shortly after their production while material M1 was produced several years before the leaching tests.

According to the safety data sheets and knowing the composition of the materials, it was possible to estimate the percentage of release of styrene for M1, M2 and M3 and furfuryl alcohol for M4. The percentage release, or release rate, indicates the mass of a substance, in this case styrene and furfuryl alcohol, released relative to the initial mass of these substances in the corresponding fresh material. Due to the confidentiality of material composition information, only the estimated (i.e. calculated) percentage release results obtained during leaching tests are presented in Table 5.

**Table 5:** Estimated percentage of release of chemicals during leaching

Chemicals	M1	M2	M3	M4
Styrene (C_8_H_8_)	0.0011%	0.0038%	0.0042%	Not present
Furfuryl alcohol (C_5_H_6_O_2_)	Not present	Not present	Not present	0.47%

As it can be seen from this table, the release of resin compounds is low compared to the total amount present in the original bio-composite. Furthermore, as styrene is a volatile compound, it is plausible that material M1, being the oldest of styrene-containing materials tested and having an initial amount of resin comparable to M2 and M3 showed a lower release of styrene in the water samples. The bio-composite material M4 was similarly analysed shortly after its manufacture, but as we only had one sample of material containing furfuryl alcohol, no conclusion could be drawn on the effect of volatilisation.

**Fig. 5:**
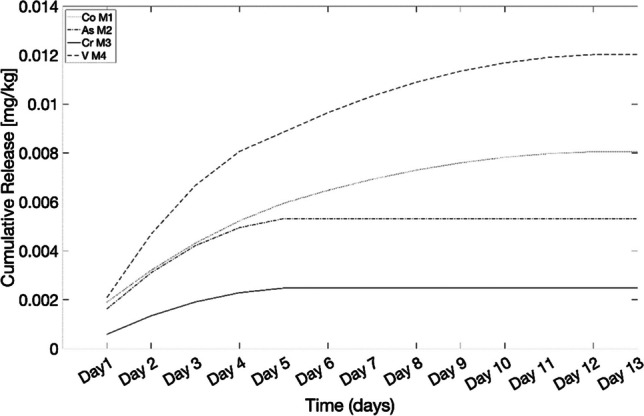
Cumulative release of Co leached from M1, As released from M2, Cr leached from M3 and V released from M4

The cumulative release curves for leached heavy metals from four analysed materials are shown in Fig. 5. As it can be seen from this figure, leaching is mainly influenced by the content of heavy metal in the bio-composite material. A deeper analysis of the cumulative release curves reveals that certain metals such as Co, Cr and V exhibit a pronounced slope across all four materials. This indicates a rapid release of the chemical (high leaching), driven by a strong dissolution force of the substance in the eluent. As time progresses (beyond one week), the curve levels out and reaches a plateau. This plateau indicates a significant slowdown in the leaching process, accompanied by a reduction in the driving force, resulting in the dissolution rate no longer being as rapid as in the initial stages. Analysing this plateau is crucial for understanding the leaching behaviour. In previous literature, van der Sloot and Kosson (2012) and Cappuyns and Swennen (2008) observed a similar trend in the release of heavy metals due to leaching. In the first study (van der Sloot & Kosson 2012; van der Sloot et al. 2017), the plateau suggested that the solution had become saturated with the solute, implying that the introduced liquid (influent) could no longer dissolve more of the substance as it had reached its maximum solubility under the prevailing conditions. In the second study, Cappuyns and Swennen (2008) observed the gradient concentration as influential parameter for the leaching behaviour. Thus, the driving force is the concentration gradient between the solution analysed for leaching and the fresh influent, reaching the chemical equilibrium at the observable plateau. In this study, concerning the leaching from the novel bio-composite materials, the influent was renewed without recirculation. Therefore, it would be inaccurate to describe the effluent solution as saturated; it probably depends only on the number of heavy metals in the bio-composite and the driving force driving the leaching is represented by the concentration gradient. More data and information are needed to perform more accurate analyses, i.e. to establish with larger accuracy why the plateau was observed in the cumulative release curves.

Figure 5 displays the cumulative release for Co in M1, As in M2, Cr in M3 and V in M4, respectively. Further details about cumulative release curve can be found in the Supplementary Material.

### Environmental risks of leaching from canal bank protection elements made of bio-composite materials

The study evaluated the effectiveness of the methodological approach to assess the leaching of heavy metals and resin compounds from novel bio-composite materials in the aquatic environment, in the absence of on-site data. This section addresses the efficacy of the proposed methodology. The laboratory leaching tests demonstrated the suitability of this approach in simulating real-world conditions, providing a reliable basis for assessing leaching behaviour of novel bio-composite materials. The tests were able to simulate two different real case scenarios: (i) stagnant conditions and (ii) advective conditions. The cost-effectiveness of employing this proposed methodology in assessing the environmental impact of these novel materials is enhanced by the possibility of predicting leaching behaviour without on-site data.

To simulate the real conditions, all cases were evaluated by considering only the first day of leaching results as the worst-case scenario. Although certain metals exhibited higher leaching values on day 2, it was decided to consider the first day as the worst-case scenario. This decision takes into account the volatile nature of styrene and furfuryl alcohol, for which the leaching tests were limited to a 24-h time range. For the heavy metals, the differences between the first day and second day were very limited. With regard to the winter and summer conditions only, the results for winter season conditions are shown, representing the worst-case scenario with elevated water levels in the canal resulting in a greater fraction of bio-composite material below the water level. Consequently, higher quantities of heavy metals and resin compounds may leach into the water from the bio-composite material under these conditions. Additional information concerning the summer season conditions can be found in the Supplementary Material.

#### Deterministic environmental risk assessment

**Fig. 6:**
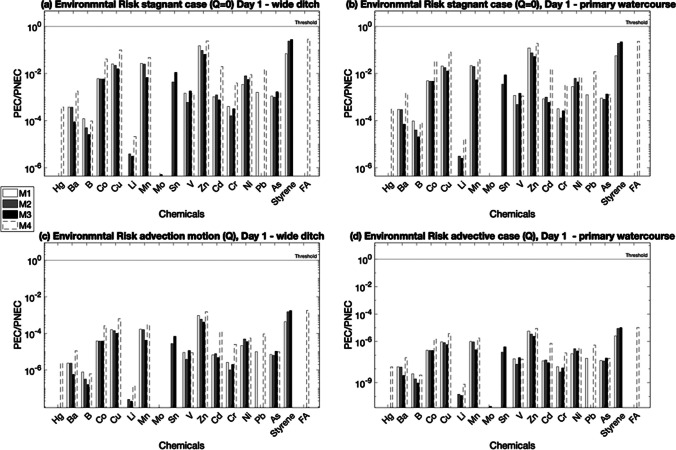
Environmental risk expressed as PEC/PNEC on day 1 for stagnant and advective conditions for both wide ditch and primary watercourse

Figure 6 shows the environmental risk assessment results on day 1 (expressed as PEC/PNEC ratios) for all bio-composite materials, for stagnant and advective flow conditions for both wide ditch and primary watercourse type canals.

As can be seen from Fig. 6, all environmental risks under all conditions are below the threshold for all four bio-composite materials.

A comparison of the results of the stagnant water and advective flow cases reveals that the stagnant case, as illustrated in Fig. 6a, b for the wide ditch, and primary watercourse, respectively, yielded higher PEC/PNEC ratios for all the chemicals analysed. This is because in the stagnant case, where there is no flow, the assumed instantaneous mixing resulted in retention of chemicals in the water without transport. Unlike this, in the advective flow case, shown in Fig. 6c for a wide ditch and Fig. 6d for a primary watercourse, the presence of a flow rate allows the transportation of chemicals, leading to further dilution. This results in lower PEC/PNEC ratios for the advective case for both the wide ditch and primary watercourse.

Further, when comparing the wide ditch results with the primary watercourse results, lower PEC/PNEC ratios were observed for the primary watercourse in both stagnant and advective cases. This can be attributed to the larger dimensions of the primary watercourse, resulting in a greater volume of water and consequently increased dilution in the primary watercourse.

The environmental risk assessment carried out in this study does not take into account the fate of chemicals released and subsequent effects, due to the lack of onsite data. For example, certain metals such as Pb, Zn and Cu exhibit significant bio-accumulative properties, while some metals can react with oxygen, leading to the formation of toxic substances (Breida et al. 2019). Furthermore, leaching behaviour is strongly influenced by the factors that represent environmental conditions, such as the pH, eluent temperature and redox conditions (Cappuyns & Swennen 2008). Given the aforementioned lack of on-site information, this study employs laboratory leaching tests to simulate real-world scenarios, focusing solely on assessing the environmental impact of using these new bio-composite materials in aquatic environments. The availability of on-site data, such as chemical analysis of water and/or sediment samples, would have provided a more complete picture of the leaching behaviour of these materials in a surface water canal. This data would have been useful for assessing not only the level of leaching but also the potential chemical reactions of leachate elements with the oxygen present in the water. This includes analysing the likelihood of the formation of toxic compounds such as oxidates, which provides critical insight into the overall environmental impact. Furthermore, on-site data is also valuable in defining the reliability of the laboratory tests carried out by comparing the results obtained with the chemical analyses carried out in the field.

#### Stochastic environmental risk assessment

The limitations associated with an environmental risk assessment based only on laboratory data were mitigated by implementing a stochastic approach. In this approach, variables such as leachate concentrations and water flow velocities were assumed uncertain. The aim was to analyse the potential impact of realistic variations in these values that could exist in real-world conditions and could be obtained through field testing rather than controlled laboratory experiments. The purpose of this analysis was to determine whether such variations could affect the overall environmental risk levels, increasing the PEC/PNEC values above the threshold of 1.00.

The stochastic assessment approach is based on the Monte Carlo method with 10,000 trials. The selection of 10,000 trials was based on the observation that no significant changes were observed when the number of iterations was increased beyond this value (not shown here). Two sensitivity cases were modelled, s1 with uncertain leachate concentrations and s2 with uncertain water velocity, both using pre-specified probability density functions, as described in ‘‘Leaching scenarios’’. The eight cases mentioned in the previous section were also analysed here. Regarding the stagnant water case, only the variations in leachate concentrations were considered, as there is no flow in that scenario. Materials M3 and M4 were chosen as representative worst-case materials as they showed the highest leaching in terms of styrene (M3) and furfuryl alcohol (M4), as can be observed from Table 5.

**Fig. 7:**
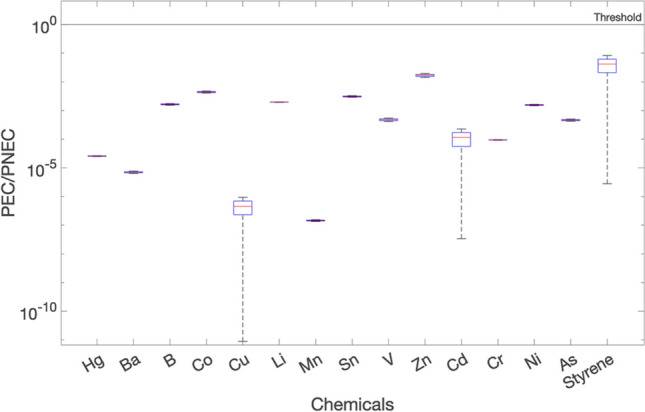
Environmental risk expressed as PEC/PNEC on day 1 for the wide ditch, stagnant case: sensitivity s1 case, for M3

**Fig. 8:**
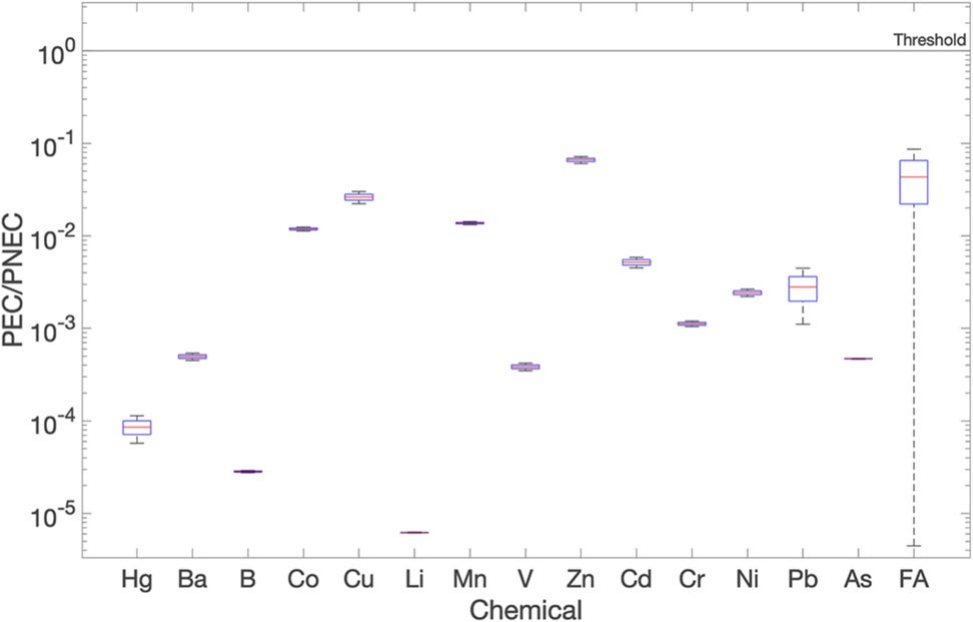
Environmental risk expressed as PEC/PNEC on day 1 for wide ditch, stagnant case: sensitivity s1 case, for M4

The results obtained for the wide ditch, stagnant water, winter condition and materials M3 and M4 are shown in Figs. 7 and 8, respectively. As can be seen from this figure, the environmental risk levels, expressed by the PEC/PNEC ratios, are all below the threshold of 1.00. The primary watercourse is characterised by a larger volume of water resulting with a higher dilution for the stagnant scenario, which significantly reduces the PEC concentrations. Consequently, the environmental risk levels expressed as PEC/PNEC ratios are even lower for the primary watercourse (results not shown).

The results obtained for the sensitivity case s1 for the wide ditch, which represents the stagnant case for M3 and M4 on day 1 (Figs. 7 and 8), showed minimal deviation from the deterministic approach (Fig. 6a). This is because the leachate concentrations were modelled using a narrow uniform distribution, aligning well with experimental data. The range of values used to simulate the uniform distribution was based on the analyses of the effluent 1 (1st column) and effluent 2 (2nd column) from the conducted leaching tests. These results of both columns demonstrated a high level of concordance and showed minimal discrepancies. Consequently, the results observed from this s1 sensitivity case matched well with the deterministic obtained results. The inclusion of field data would have further improved the sensitivity analysis, providing the chance to consider a wider range of values and thus a more complete evaluation of potential variations in the results.

**Fig. 9:**
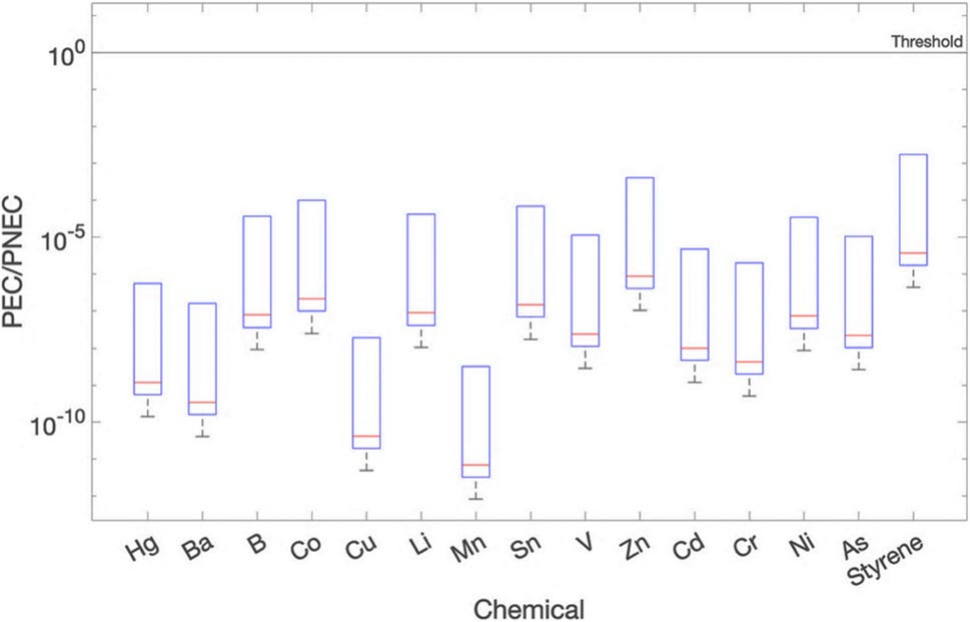
Environmental risk expressed as PEC/PNEC for advective flow on day 1, wide ditch: sensitivity s2 case for M3

**Fig. 10:**
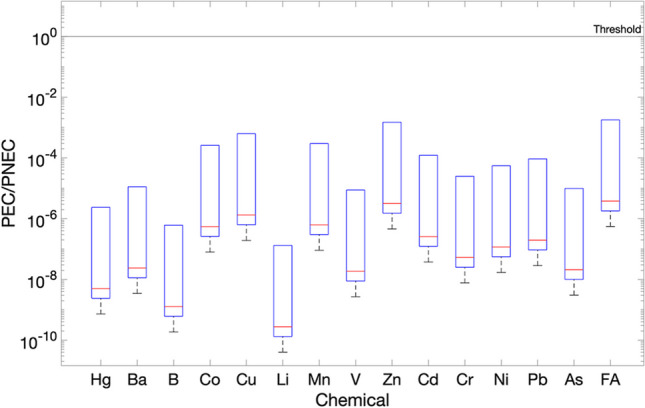
Environmental risk expressed as PEC/PNEC for advective flow on day 1, wide ditch: sensitivity s2 case for M4

The results for sensitivity case s2 (different water velocities at fixed leachate concentrations) in the advective scenario for wide ditch and are shown in Figs. 9 and 10, for M3 and M4, respectively. The water velocity was modelled by using normal distributions as described in ‘‘Leaching scenarios’’.

As can be seen from Figs. 9 and 10, the results obtained indicate a significant variation in the PEC/PNEC values over a range of 10E-09 to 10E-02 and precisely a PEC/PNEC ratio about 0.108 for furfuryl alcohol in M4, as observed in Fig. 10. This can be attributed to the changes in the water velocities. The maximum values of PEC/PNEC ratios were obtained at minimum water velocity, for the reasons mentioned before.

The sensitivity analysis indicates that water velocity, and therefore flow rate, is the most influential input environmental parameter affecting the outputs. As expected, higher flow rates increase dilution and lead to lower PEC values. This is consistent with the results of the deterministic approach and further confirms that stagnant conditions (no flow) combined with varying leachate concentrations represent the worst-case scenario.

## Conclusions

The objective of this paper was to develop a methodology and present associated results obtained for the environmental risk assessment associated with the potential leaching of toxic substances from four new types of bio-composite materials used in the production of canal bank protection elements. A comprehensive set of laboratory leaching tests was carried out to generate input data for this assessment. Eight cases of potential leaching were analysed by performing the risk analyses for two flow conditions (stagnant water and advective flow) in two types of canals (wide ditch and primary watercourse) and for two flow rates (low and high, corresponding to summer and winter conditions, respectively). Both deterministic and stochastic environmental risk assessment approaches were used, the former to assess the environmental risks and the latter to assess the sensitivity of the results obtained in the deterministic case due to uncertainties in leachate concentrations and water velocities (i.e. flow rates).

The leaching tests yielded results that could be used as input for the environmental risk assessment. The results indicated that the release of toxic substances remains within acceptable limits, as the PEC/PNEC ratios did not exceed the environmental threshold of 1.00 in all cases analysed. However, under stagnant conditions in the wide ditch, certain chemicals showed slightly higher PEC/PNEC ratios compared to the other cases. Specifically, furfuryl alcohol for material M4 had the highest PEC/PNEC ratio of 0.108, which was closest to the threshold of 1.00. Zn was the second chemical for material M4, approaching the threshold of 1.00 under stagnant conditions with a PEC/PNEC ratio of 0.00898. For materials M1, M2 and M3, Zn and styrene showed the highest PEC/PNEC values in stagnant scenarios. The sensitivity analysis performed in this study indicated that water velocity and thus flow rate is the most influential input parameter affecting the outputs.

Overall, the findings obtained show the importance of monitoring and managing aforementioned chemicals, especially under specific environmental conditions, to ensure the protection of the ecosystem. It would also be interesting to assess the potential risks to human health from the use of these new bio-composite materials, for example, by analysing human contact with water during recreational activities such as swimming in the canals. However, this assessment is out of the scope of this paper, i.e. this remains to be done as part of future work.

The obtained results are only indicative at this stage, as these are based on laboratory leaching tests and a number of assumptions mentioned in ‘‘Environmental risk assessment’’. To further validate the accuracy of the approach of combining laboratory leaching tests with environmental risk assessment, additional field tests are required to collect water and sediment samples from the canal where the bank protection elements are located over a longer period of time. Analysis of these field samples will provide an overview of the water quality and also the actual leaching of toxic substances. Furthermore, these field tests would provide valuable information on the effects of dilution in the real-case scenarios, which is a significant parameter in determining whether the leachate concentration of a specific chemical is within the environmental risk limits. In addition to field tests, simulation of flow rate conditions, particularly ‘almost no flow’ conditions, could be improved by using more detailed mixing models rather than the assumed instantaneous mixing used in this work.

Despite the above considerations, the integration of leaching tests with the environmental risk assessment methodology, as outlined in this study, has proven its effectiveness in assessing the risks associated with the use of new bio-composite materials. This combined approach is significant for its applicability to various other implementations of bio-composite materials, ranging from building façade elements to water level scales installed in canals.

### Supplementary Information

Below is the link to the electronic supplementary material.

Supplementary file1 (DOCX 3754 KB)

## Data Availability

All available data are provided in both the manuscript and the supplementary information file.

## Declarations

### Disclaimer

This paper reflects only the author’s view, the Commission is not responsible for any use that may be made of the information it contains.
