# Supplementary material for: Environmental risk assessment related to using resource recovery-based bio-composite materials in the aquatic environment with new laboratory leaching test data
Source: Environ Sci Pollut Res Int. 2024 Feb 21;31(14):21057–72. doi: 10.1007/s11356-024-32522-8 (PMC10948581; doi:10.1007/s11356-024-32522-8)
Supplement: Supplementary file 1 — Supplementary file1 (DOCX 3754 KB) [file 11356_2024_32522_MOESM1_ESM.docx]

**Environmental Risk Assessment of the use of water-resource based bio-composite materials in aquatic environments by using laboratory leaching tests: Supplementary Material**

# Primary watercourse profile


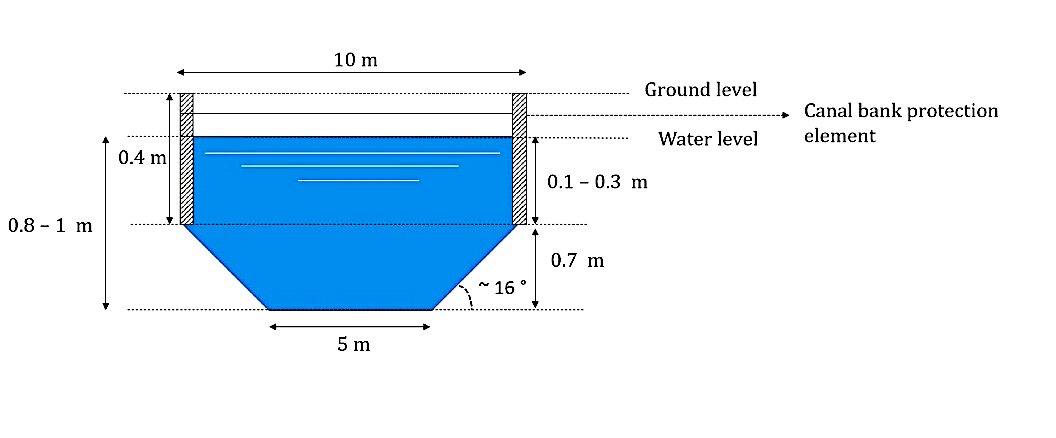
 The primary watercourse has a similar profile as the wide ditch with a trapezoidal geometry, characterised by larger dimensions that vary in cross-section and side slope. Details of the primary watercourse profile are presented in Figure 1.

Figure 1. Primary watercourse profile

# Summer season conditions results

The summer season condition is characterized by a lower water level (0.1 m) due to the dry climate. As a result, the cross-sectional area of the water is reduced, leading to a lower volume of water and low flow rate. In addition, the bio-composite canal bank protection element is partially submerged in the water, resulting in less release of chemicals into the surface water. Under summer conditions, the PEC/PNEC ratio decreases, leading to a reduction in environmental risk.

The obtained results for the summer season conditions are presented in this supplementary material for both deterministic and stochastic approaches.

## Deterministic approach

### Stagnant case

The stagnant case is characterised by the absence of flow rate, which extends the residence time of chemicals in surface water, and limits their transport. Under these conditions, the observed PEC/PNEC ratios are higher and indicate a high environmental risk. However, as mentioned above, under summer conditions the PEC/PNEC ratios are reduced compared to the winter conditions (characterised by higher flow and water level).


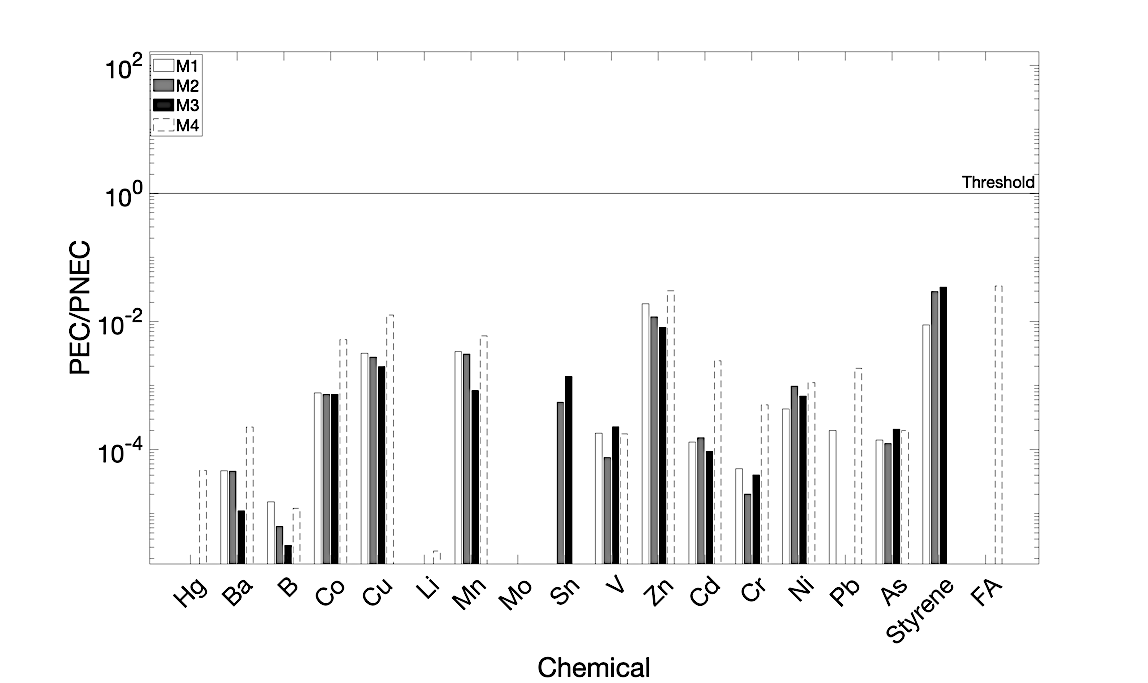
The obtained results for all four bio-composite alternatives for both wide ditch and primary watercourse are shown in Figures 2 - 3.

Figure 2. Stagnant case, wide ditch under summer season conditions.


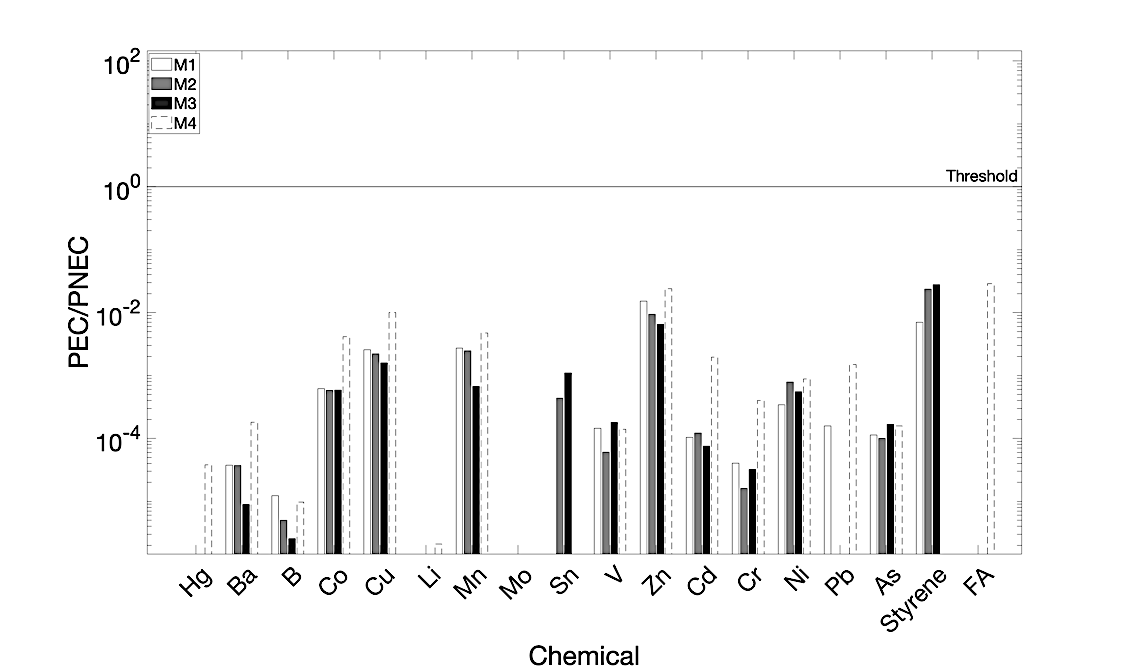


Figure 3. Stagnant case, primary watercourse under summer season conditions.

### Advective flow case

The presence of flow rate (advective flow case) increases the dilution due to the periodic renewal of the water volume per day. This results in a lower PEC/PNEC ratio, especially under summer season conditions, for the reasons explained above. Results for the advective flow case for all four bio-composite materials, for both the wide ditch and the primary watercourse under summer conditions, are shown in Figure 5 - 6.


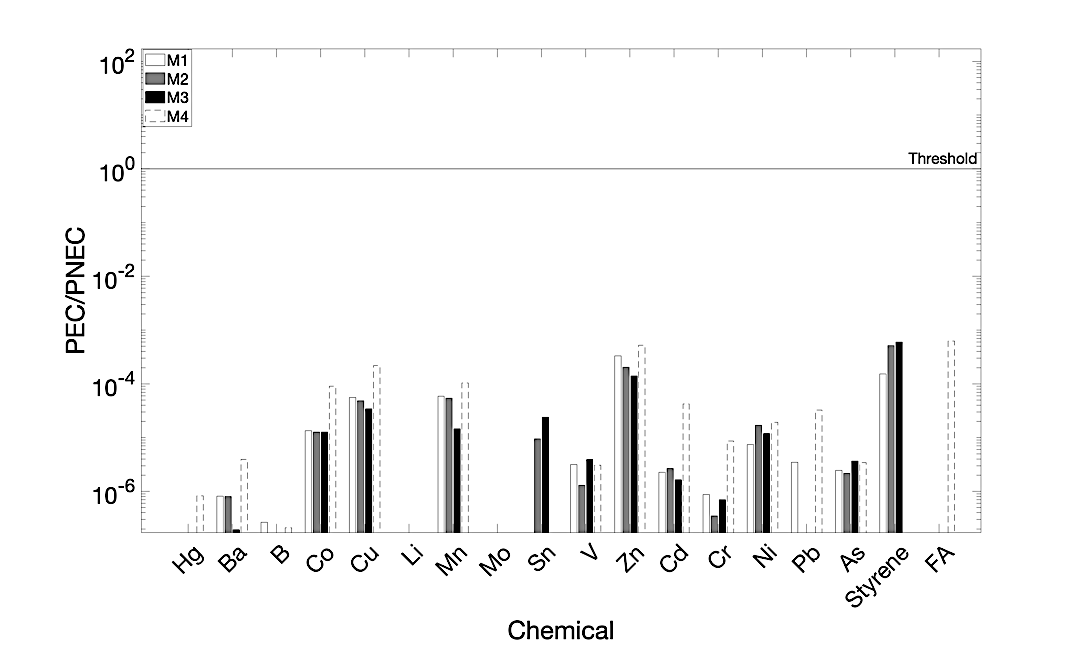


Figure 4. Advective flow case, wide ditch under summer season conditions.


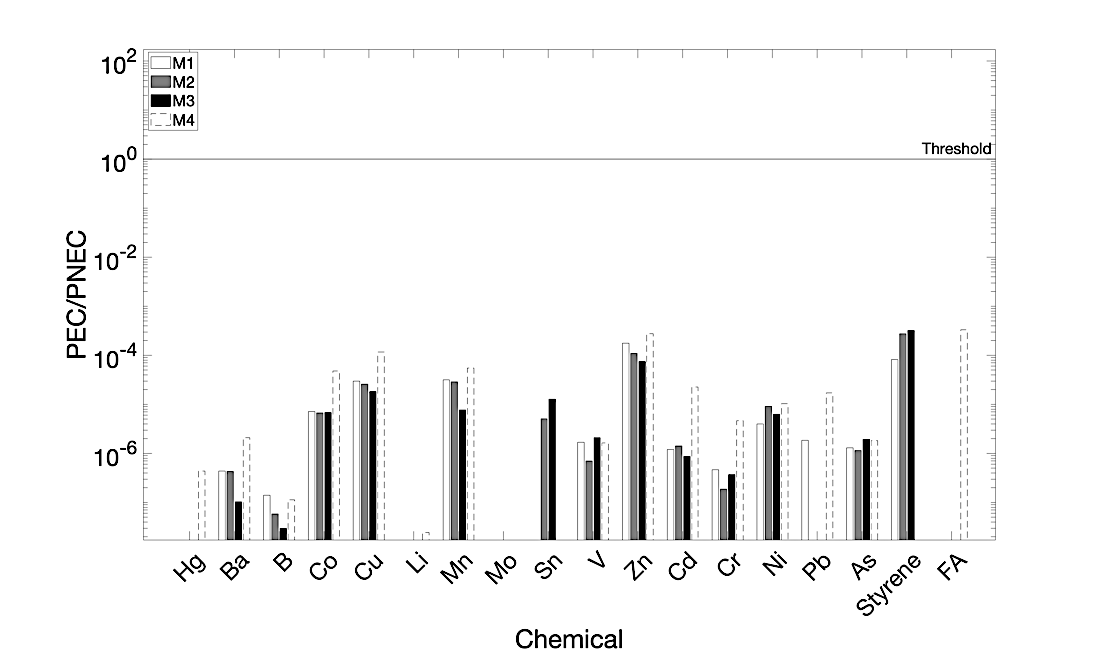


Figure 5. Advective flow case, wide ditch at low flow conditions.

## Stochastic approach

### Stagnant case: sensitivity analysis s0 (variation of flow rate) for M3 and M4 for both wide ditch and primary watercouse.

The sensitivity analysis used a stochastic approach, specifically using the Monte Carlo method with 10,000 trials. In the first sensitivity case (s1), the concentrations from the laboratory leaching tests were simulated using the uniform distribution under stagnant conditions (no flow). For this analysis, M3 and M4 were chosen as relevant materials due to the high release of styrene and furfuryl alcohol, respectively.


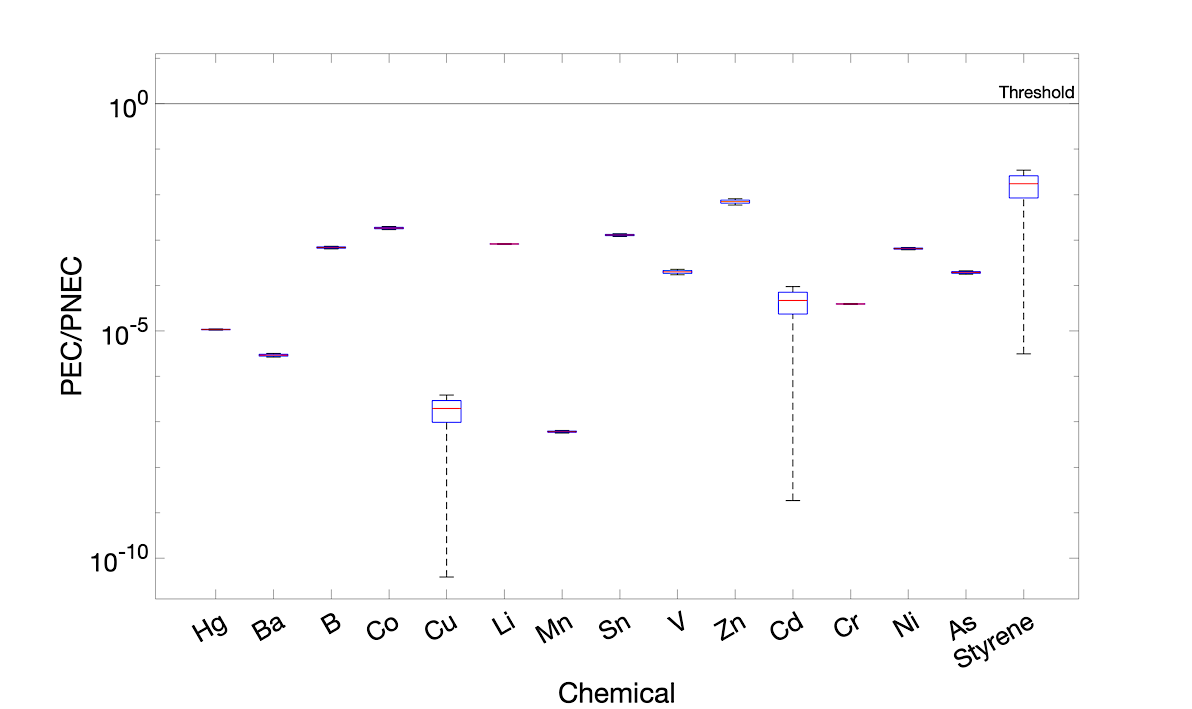
Results for both wide ditch and primary watercourse under summer conditions for M3 and M4 are displayed in Figure 6 - 9.

Figure 6. Stagnant case condtitions, M3 wide ditch under summer conditions.


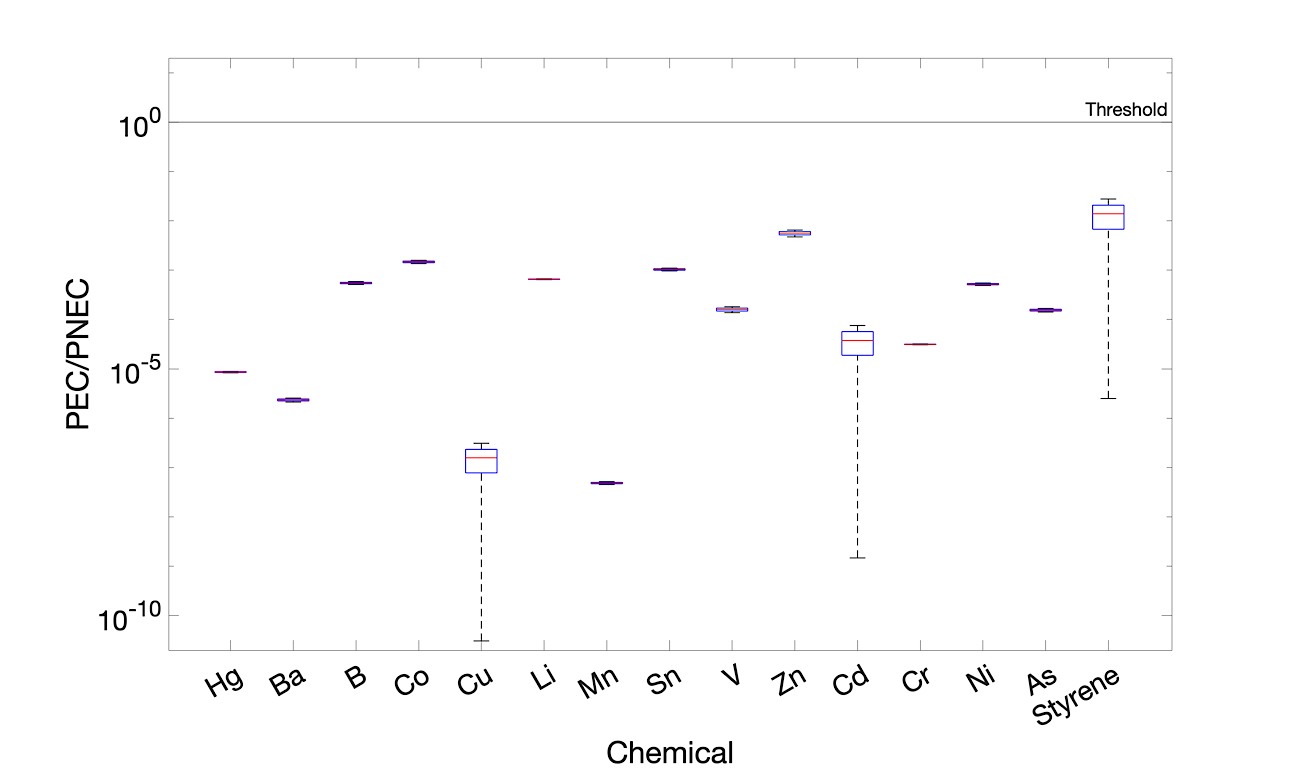


Figure 7. Stagnant case condtitions, M3 primary waterflow under summer conditions.


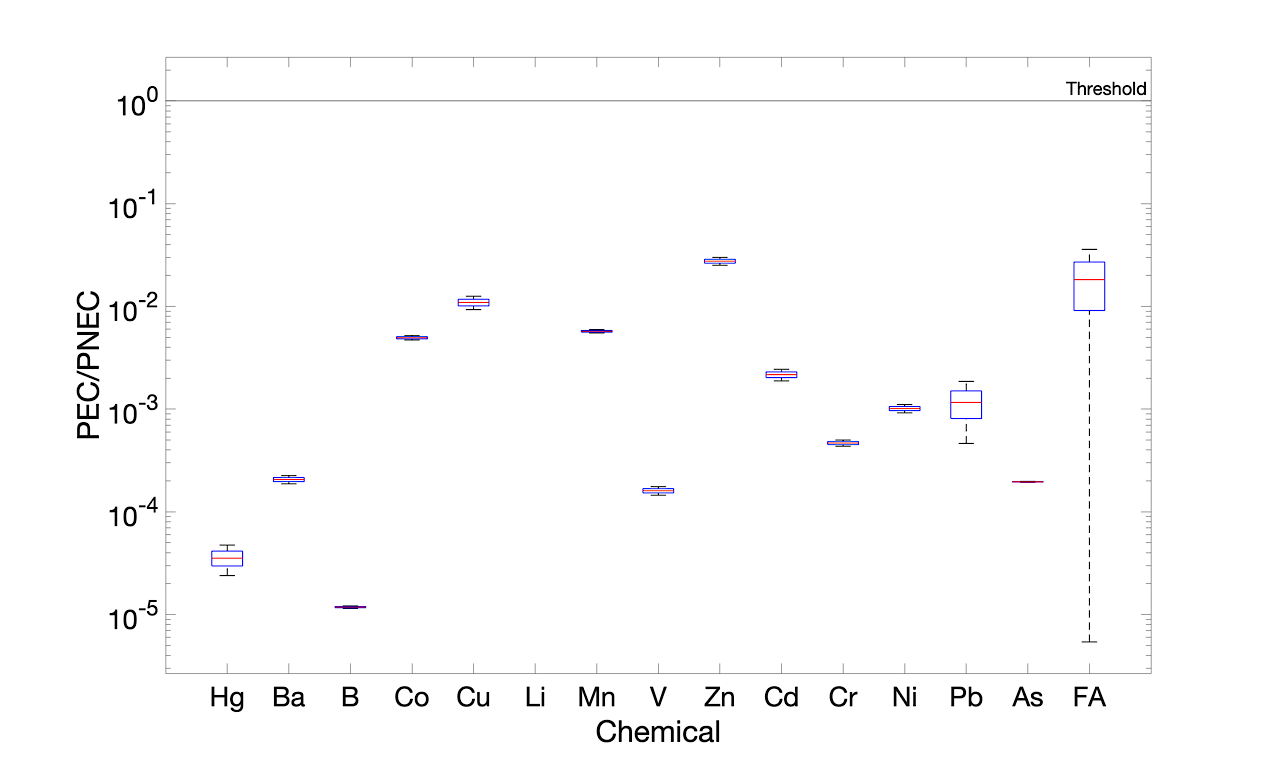


Figure 8. Stagnant case condtitions, M4 wide ditch at low flow conditions.


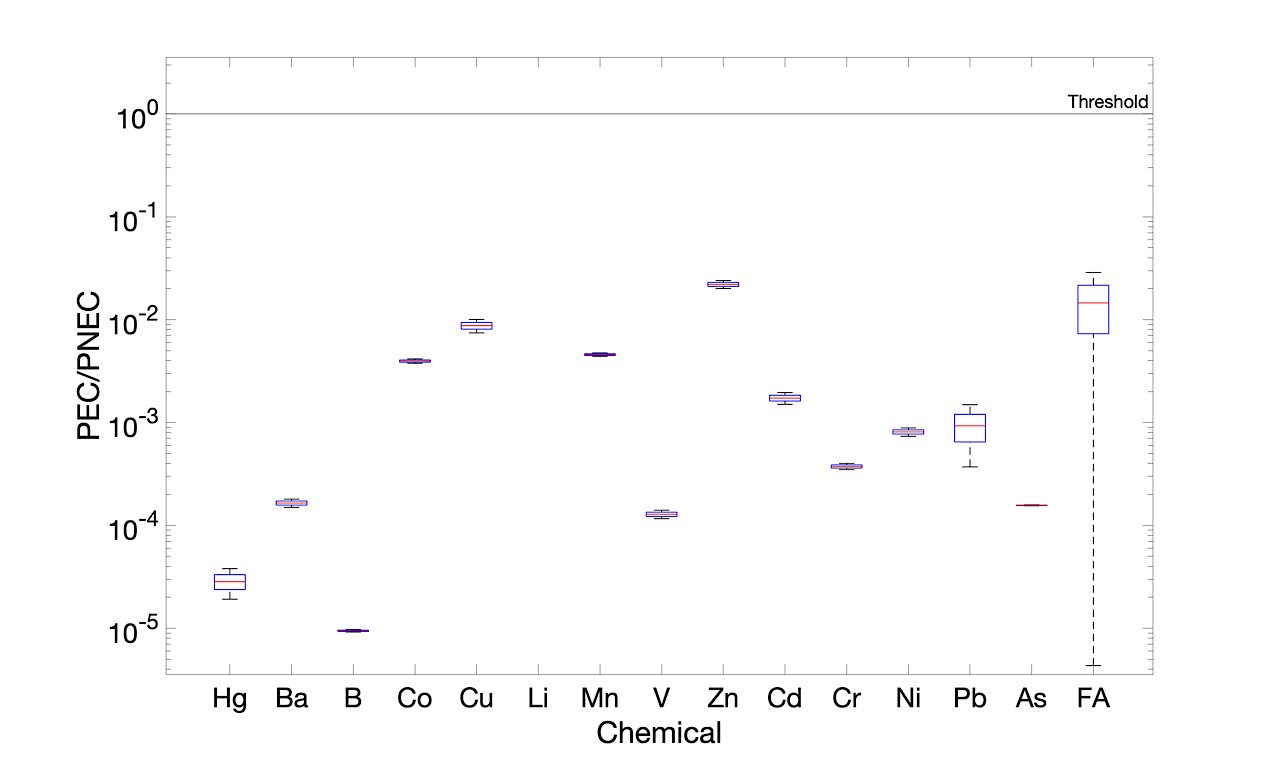


Figure 9. Stagnant case condtitions, M4 primary waterflow under summer conditions.

### Advective case: sensitivity analysis s2 (variation of flow rate) for M3 and M4 for both wide ditch and primary watercouse.

Results for M3 and M4 (based on above) for both wide ditch and primary watercourse under low flow conditions, are shown in Figure 10 - 13.


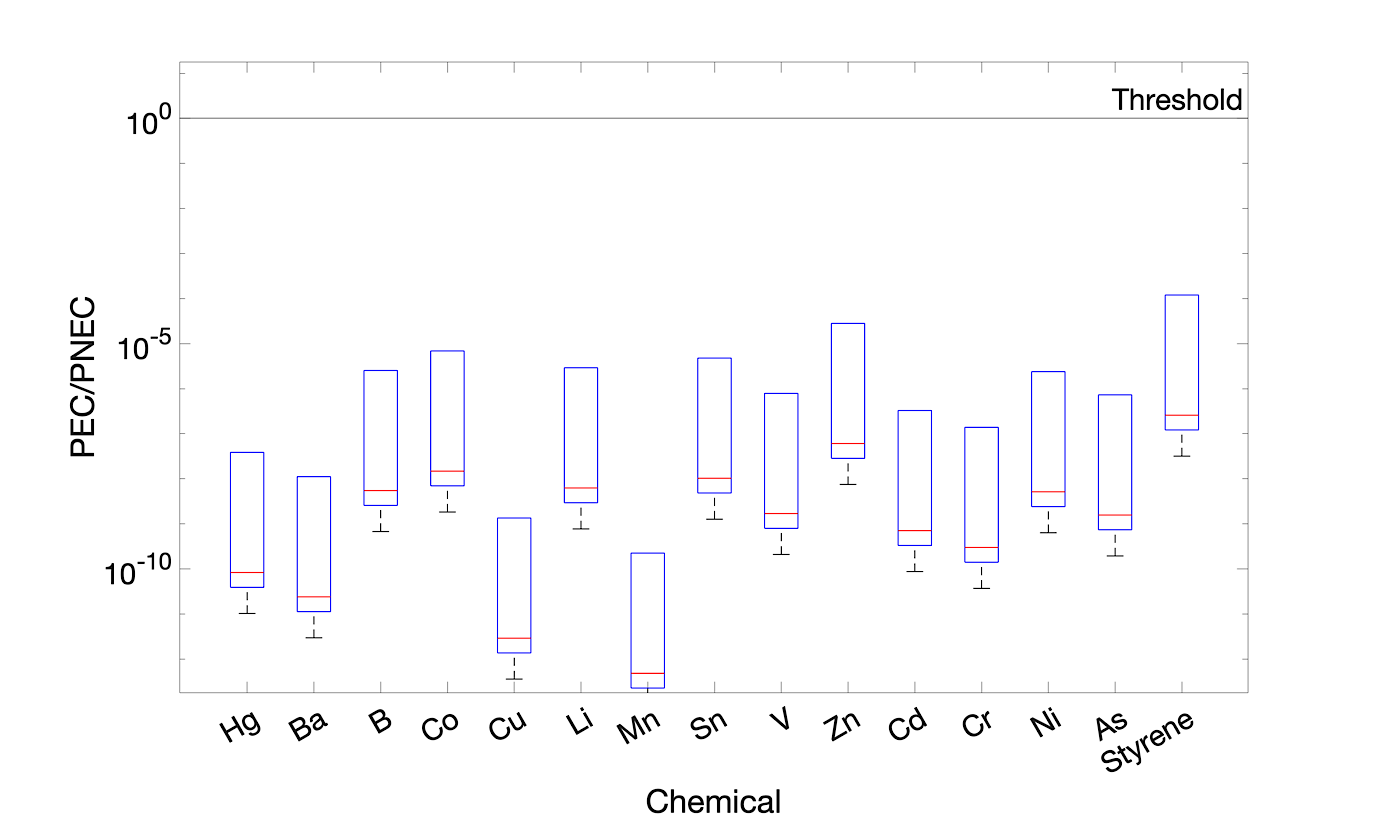

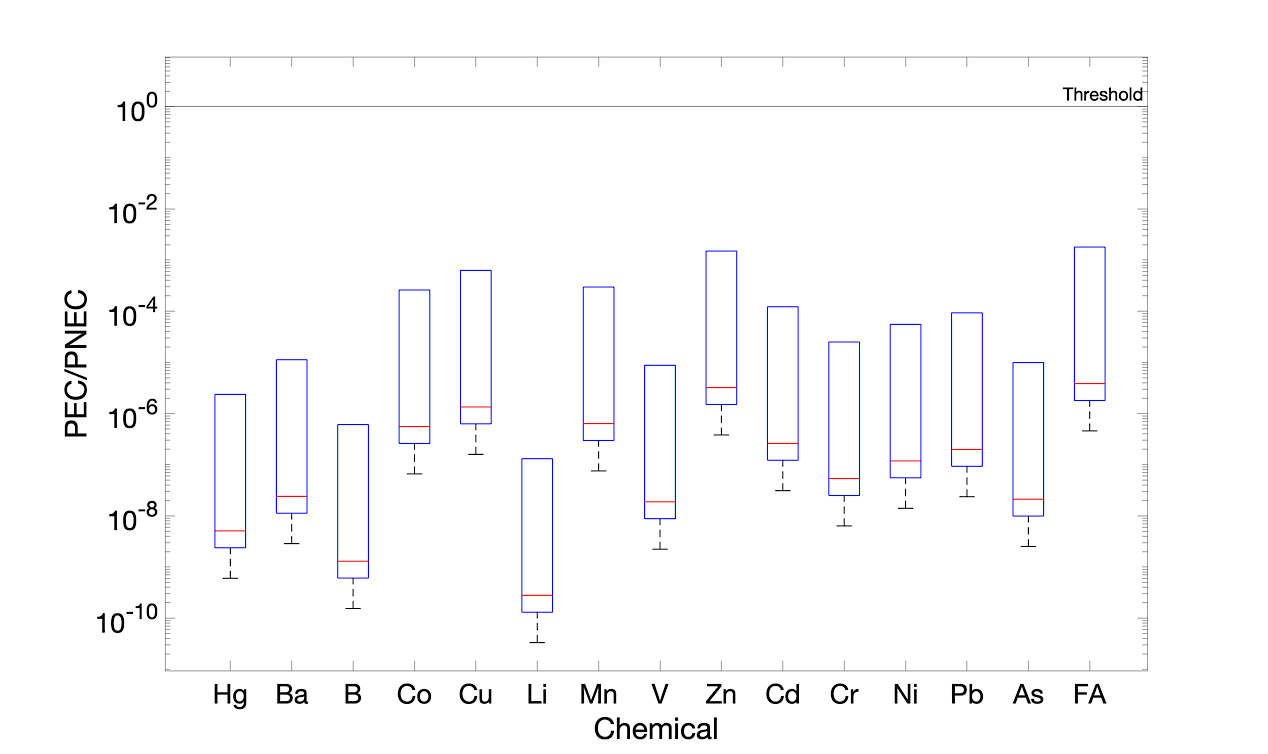

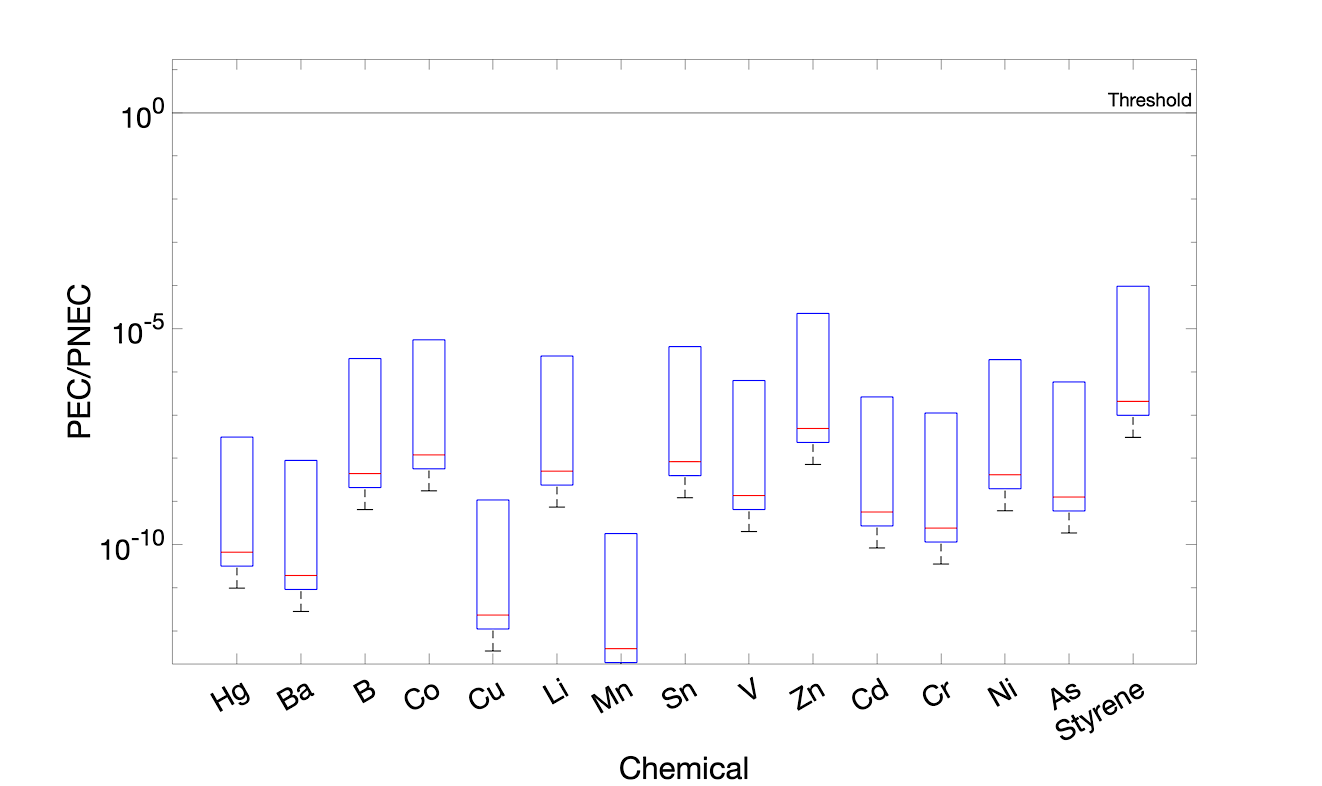


Figure 10. Advective flow case condtition, M3 wide ditch under summer conditions.

Figure 11. Advective flow case condtition, M3 primary waterflow under summer conditions.


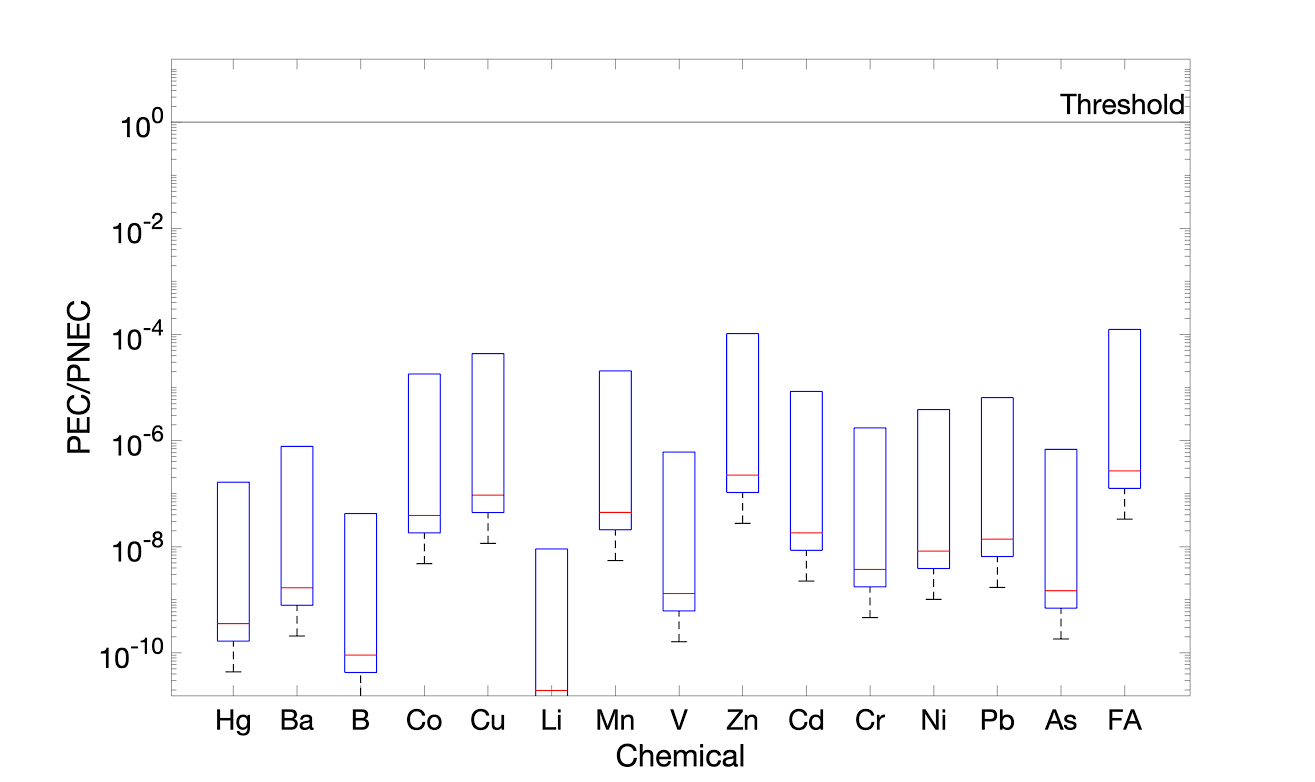


Figure 12. Advective flow case condtition, M4 wide ditch under summer conditions.


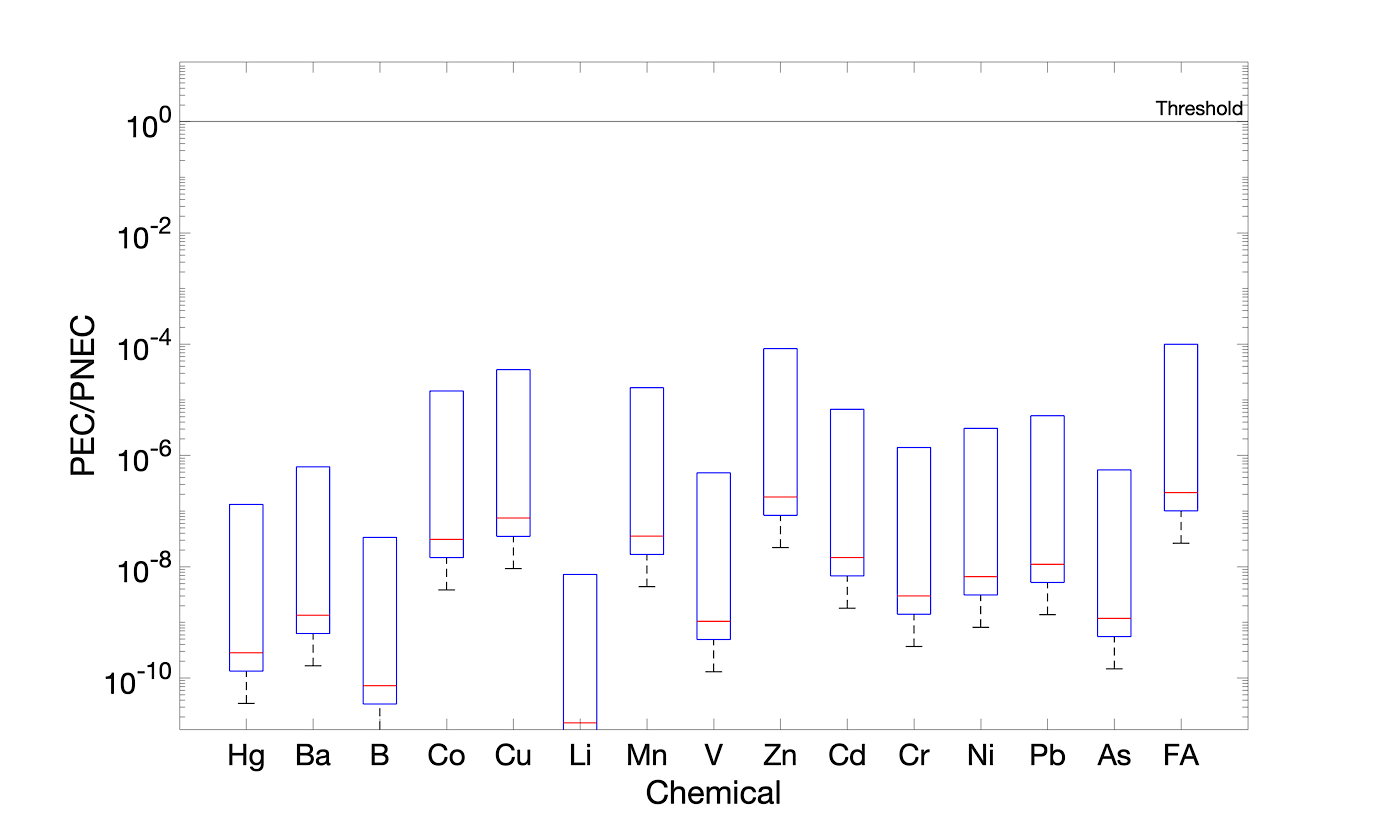


Figure 13. Advective flow case condtition, M4 primary watercourse under summer conditions.

# Cumulative release rate

Four samples corresponding to four different days were analysed from the leaching tests: day 1, day 2, day 6 and day 13. To ensure complete information, linear interpolation was used to determine the leaching values for the missing data points. Cumulative concentrations up to day 13 were then calculated. The leaching results, initially expressed in mg/l, were then converted to mg/kg product concentrations using the following equation.

| $C_{mg/kg}=\frac{C_{L}*V_{L}}{M_{L}}$ | (1) |
| --- | --- |

Where:

- $C_{mg/kg}$ is the equivalent concentration of the leaching in mg per kg of material released from leaching conditions in 1 day [mg/kg].
- $C_{L}$ is the leaching concentration in one day [mg/l].
- $V_{L}$ is the volume of water added in one day of leaching (l).
- $M_{L}$ is the mass of the material used for leaching test (0.3 kg).

Linear interpolation was used to estimate the intermediate time points between day 1 and day 13. The cumulative curve was constructed by calculating all time points within this interval. At day 13, the results represent the total mass released by 0.3 kg of material under laboratory leaching conditions. The results obtained were then evaluated in terms of potential release in a 1 m compartment of the canal using the following formula for summer and winter conditions.

| $C_{R,s}=\frac{C_{mg/kg}*M_{sub,s}}{V_{w,s}}$ | (2) |
| --- | --- |
| $C_{R,w}=\frac{C_{mg/kg}*M_{sub,w}}{V_{w,w}}$ | (3) |

Where:

- $C_{R,s}$and $C_{R,w}$are the new concentrations released in one day from the canal bank protection over 1 m of length of bio-compoiste canal bank protection under summer and winter conditions, respectively [mg/m3].
- $C_{mg/kg}$ is the equivalent concentration in mg/kg released in one day under laboratory leaching test conditions, calculated in Eq. (1) [mg/kg].
- $M_{sub,s}$ and $M_{sub,w}$ are the masses of subermeged canal bank protection over 1 m length, under summer and winter conditions respectively [kg].
- $V_{w,s}$and $V_{w,w}$ are the volume of water over 1 m of length of bio-composite canal bank protection element under summer and winter conditions respectively [m3].

The new calculated concentrations represent the Predicted Effects Concentration (PEC) over a 13-day period for a canal bank protection element covering a length of 1 m. These values were then compared with the Predicted No Effects Concentration (PNEC) collected to assess the environmental risk after 13 days. The results indicate that the PEC/PNEC ratio for all four bio-composite alternatives is below the threshold. Given this, the results presented refer to the worst case scenario, characterised by stagnant conditions and a large ditch. It was decided not to include the results for the primary watercourse and the advective scenario, as the PEC/PNEC ratios in these cases would be even lower than those presented here, falling consistently below the safety threshold of 1.00.


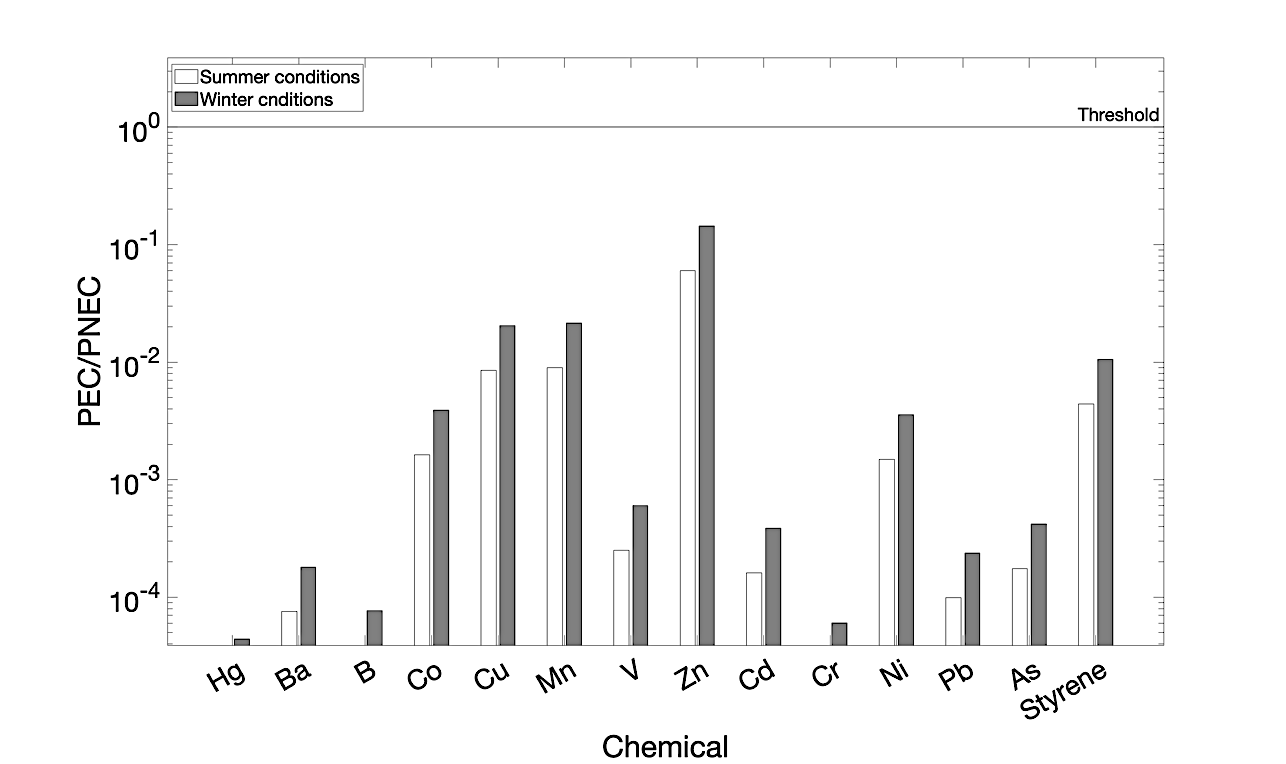
Figure 14 - 17 show the environmental risk in terms of PEC/PNEC values over thirteen days under both summer and winter season conditions.

Figure 14. Cumulative Environmental risk for M1 expressed as PEC/PNEC on day 13, stagnant case under summer and winter conditions for wide ditch.


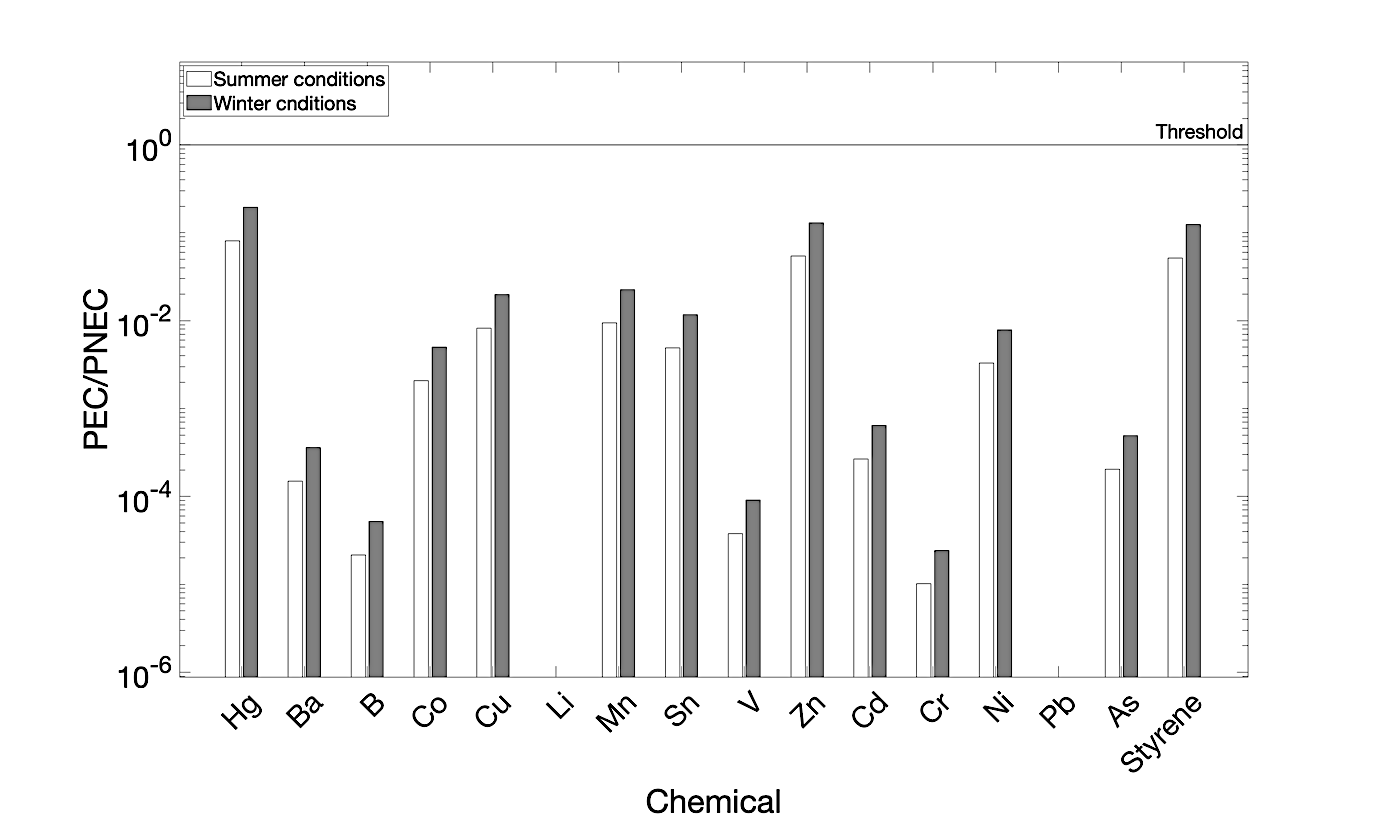


Figure 15. Cumulative Environmental risk for M2 expressed as PEC/PNEC on day 13, stagnant case under summer and winter conditions for wide ditch.


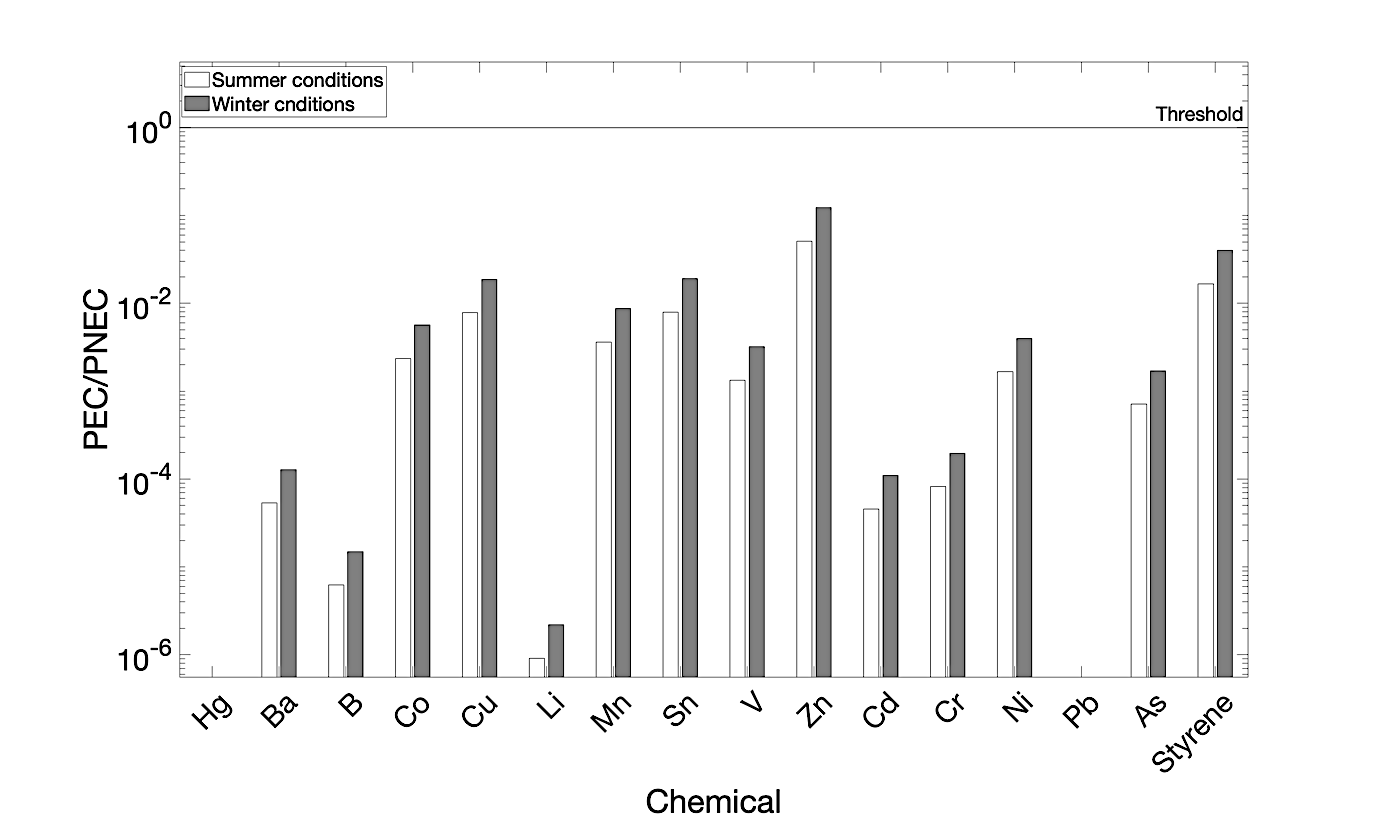


Figure 16. Cumulative Environmental risk for M3 expressed as PEC/PNEC on day 13, stagnant case under summer and winter conditions for wide ditch.


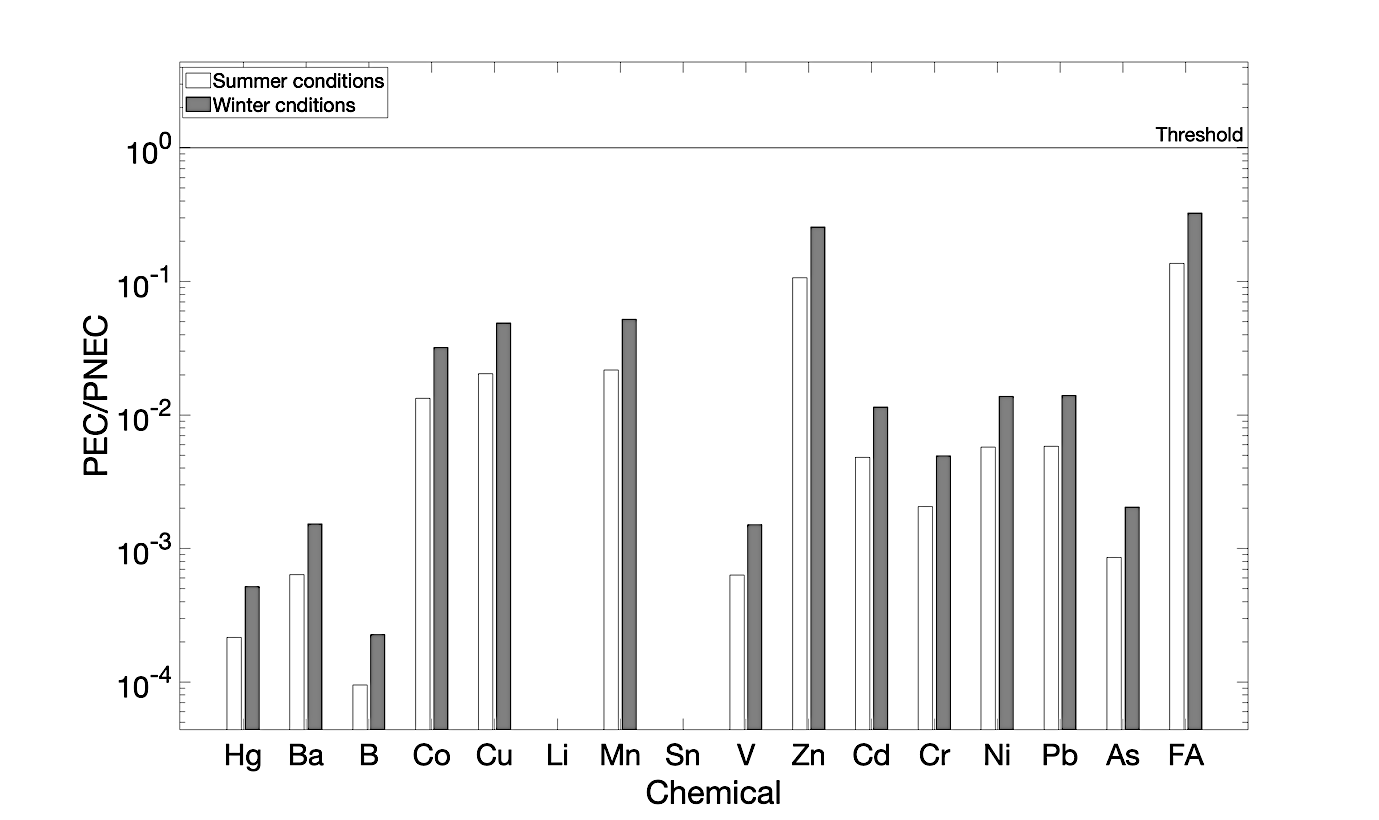


Figure 17. Cumulative Environmental risk for M1 expressed as PEC/PNEC on day 13, stagnant case under summer and winter conditions for wide ditch.
